# Nuclear ARRB1 induces pseudohypoxia and cellular metabolism reprogramming in prostate cancer

**DOI:** 10.15252/embj.201386874

**Published:** 2014-05-16

**Authors:** Vincent Zecchini, Basetti Madhu, Roslin Russell, Nelma Pértega-Gomes, Anne Warren, Edoardo Gaude, Joana Borlido, Rory Stark, Heather Ireland-Zecchini, Roheet Rao, Helen Scott, Joan Boren, Charlie Massie, Mohammad Asim, Kevin Brindle, John Griffiths, Christian Frezza, David E Neal, Ian G Mills

**Affiliations:** 1 Department of CRUK, CRUK Cambridge Institute, University of CambridgeCambridge, UK; 2 Life and Health Sciences Research Institute, School of Health Sciences, University of MinhoBraga, Portugal; 3 Department of Pathology, University of CambridgeCambridge, UK; 4 Medical Research Council Cancer Cell Unit, Hutchison/MRC Research Centre, University of CambridgeCambridge, UK; 5 Prostate Cancer Research Group, Centre for Molecular Medicine Norway (NCMM), Nordic EMBL Partnership, University of Oslo and Oslo University HospitalOslo, Norway; 6 Department of Cancer Prevention and Urology, Institute of Cancer Research and Oslo University HospitalOslo, Norway

**Keywords:** Adaptor, hypoxia, metabolism, prostate, transcription

## Abstract

Tumour cells sustain their high proliferation rate through metabolic reprogramming, whereby cellular metabolism shifts from oxidative phosphorylation to aerobic glycolysis, even under normal oxygen levels. Hypoxia-inducible factor 1A (HIF1A) is a major regulator of this process, but its activation under normoxic conditions, termed pseudohypoxia, is not well documented. Here, using an integrative approach combining the first genome-wide mapping of chromatin binding for an endocytic adaptor, ARRB1, both *in vitro* and *in vivo* with gene expression profiling, we demonstrate that nuclear ARRB1 contributes to this metabolic shift in prostate cancer cells via regulation of HIF1A transcriptional activity under normoxic conditions through regulation of *succinate dehydrogenase A* (*SDHA*) and *fumarate hydratase* (*FH*) expression. ARRB1-induced pseudohypoxia may facilitate adaptation of cancer cells to growth in the harsh conditions that are frequently encountered within solid tumours. Our study is the first example of an endocytic adaptor protein regulating metabolic pathways. It implicates ARRB1 as a potential tumour promoter in prostate cancer and highlights the importance of metabolic alterations in prostate cancer.

## Introduction

Beta-arrestin1 (ARRB1) is a ubiquitously expressed adaptor protein with a wide range of cellular and molecular functions (Lefkowitz & Shenoy, 2005). Recently, it has been shown to contribute to a number of diseases, including cancer (Dasgupta *et al*, 2006, 2011; Rosano *et al*, 2009; Liu *et al*, 2011; Lundgren *et al*, 2011). About a decade ago, a landmark study brought to light a novel nuclear role for ARRB1 in the regulation of gene transcription (Kang *et al*, 2005). Since then, other studies have confirmed this nuclear function and described its contribution to tumour growth, invasion and metastasis in lung and breast carcinoma cell lines (Dasgupta *et al*, 2011; Shenoy *et al*, 2004).

One such study showed ARRB1 to co-localise and physically interact with hypoxia-inducible factor 1A (HIF1A) in the nucleus of breast cancer cells to potentiate HIF1-dependent transcription, thereby mediating metastatic growth of breast cancer cells (Shenoy *et al*, 2004). Under normoxic conditions, HIF1A is hydroxylated at specific proline residues by prolyl hydroxylases (PHDs), tagging it for ubiquitination and subsequent degradation by the proteasome pathway (Maxwell *et al*, 1999). Hypoxia inhibits prolyl hydroxylation resulting in stabilisation of HIF1A upon which it can translocate into the nucleus and heterodimerise with HIF1B to form a functional transcription factor (TF) that binds to specific promoter regions to activate the transcription of its target genes (Semenza, 2007b). Hypoxic stabilisation of HIF1A induces a switch in cellular metabolism via transcriptional activation of a plethora of metabolic genes that results in increased glycolysis and reduced mitochondrial function (Semenza, 2007c, 2010). This metabolic reprogramming, termed the Warburg effect, allows cancer cells to meet the increase in biomass production that is required to sustain their rapid proliferation.

Based on the report that ARRB1 interacts with and regulates HIF1A activity in breast cancer cells and given the important role of HIF1A on metabolism and the critical role played by HIF1A in the progression of prostate cancer (Park *et al*, 2012), we have carried out a detailed study to determine the role of ARRB1 in prostate cancer cells using genomics and metabolomics. Herein, we report the first cistrome and transcriptome data for an endocytic adaptor. We identify a nuclear interaction between ARRB1 and HIF1A in prostate cancer cells and demonstrate that ARRB1 is recruited to promoter regions of metabolic genes in a HIF1A-dependent manner where it contributes to metabolic genes expression as a co-regulator of HIF1A transcriptional activity. We go on to show that ARRB1 acts as a modulator of cellular metabolism that facilitates glucose uptake and glycolysis and that, through modulation of TCA cycle metabolites, it induces HIF1A stabilisation under normoxic conditions, a process named pseudohypoxia. Our study implicates ARRB1 as a regulator of metabolism in prostate cancer cells.

## Results

### ARRB1 is upregulated and nuclear in prostate cancer

The *ARRB1* gene maps to the chromosome locus 11q13, which is often amplified in human cancers (Schwab, 1998; Kenny *et al*, 1999; Buchanan *et al*, 2006) (Supplementary Fig S1A). This region shows regional gain or amplification in 15% of prostate cancers (El Gedaily *et al*, 2001), and recent studies of 11q13 revealed multiple independent loci associated with risk of prostate cancer (Zheng *et al*, 2009; Chung *et al*, 2011). In addition, we found ARRB1 to be in the top 1% overexpressed genes in prostate carcinoma compared to normal tissue in a recent clinical gene expression study (Wallace *et al*, 2008) (Supplementary Fig S1B and C). Despite this, there have been no studies on the involvement of ARRB1 in prostate cancer.

**Figure 1:**
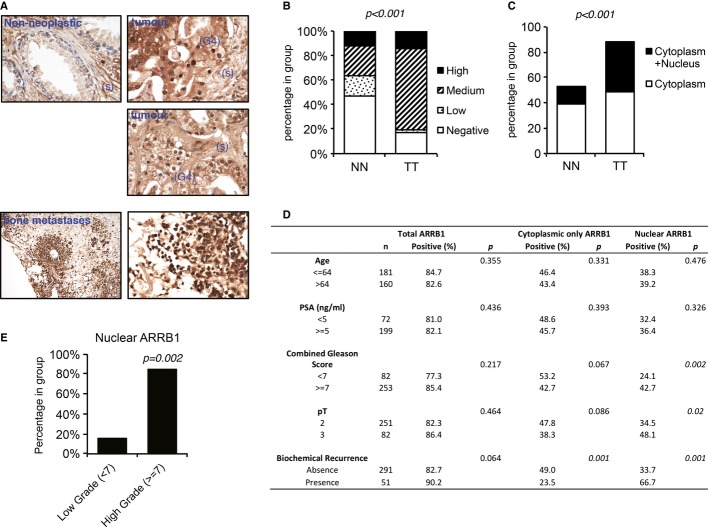
Nuclear ARRB1 is increased in prostate cancer Representative expression pattern of ARRB1 in non-neoplastic and malignant prostate cancer tissues. Non-neoplastic tissue shows weak nuclear and moderate cytoplasmic staining in luminal and basal cells. Staining is also present in stromal cells (s). Moderate to intense cytoplasmic and intense nuclear staining is noted in Gleason 4 (G4) areas of the tumour. Intense staining is noted in scattered bone metastatic prostate cancer cells.Quantification of ARRB1 staining in non-neoplastic and malignant prostate tissue shown in (A) (Porto TMA, see Supplementary information for details). NN=non-neoplastic, TT=tumour tissue. *P *< 0.001 for total positive ARRB1 cases in TT versus NN.Nuclear (solely nuclear + cytoplasmic and nuclear) or solely cytoplasmic ARRB1 staining in non-neoplastic and tumour tissue. *P *< 0.001 for positive nuclear ARRB1 in TT versus NN.Assessment of association between ARRB1 expressions (total expression versus only cytoplasmic versus nuclear) in prostate cancer samples and clinicopathological data. The comparisons were examined for statistical significance using Pearson's chi-square (χ^2^) test, *P *< 0.05 being the threshold for significance.Distribution of nuclear ARRB1 in low (< 7) and high (≥ 7) grade tumours.

Using two independent tissue microarrays (TMAs) of human prostate cancer, we examined the levels of ARRB1 protein immunohistochemically in non-neoplastic and cancer tissue (Fig1A and Supplementary Fig S1D and E). Although cytoplasmic staining was present both in non-neoplastic and tumour tissue, it showed stronger overall intensity in the tumour tissue in both TMAs (Fig1B and Supplementary Fig S1F). Importantly, the additional presence of nuclear staining was significantly higher in tumour tissue (Fig1C), and strong nuclear ARRB1 was seen in high-grade areas of the tumours (Fig1A and Supplementary Fig S1E). In addition, increased levels of nuclear ARRB1 correlated with Gleason score, stage and biochemical recurrence, suggesting an association with more aggressive disease (Fig1D and E). Consistent with previous reports of a role in invasion and metastasis (Buchanan *et al*, 2006; Shenoy *et al*, 2012), ARRB1 was also present in secondary bone metastases (Fig1A).

### ARRB1 expression levels correlate with the neoplastic phenotype of prostate cancer cells

**Figure 2:**
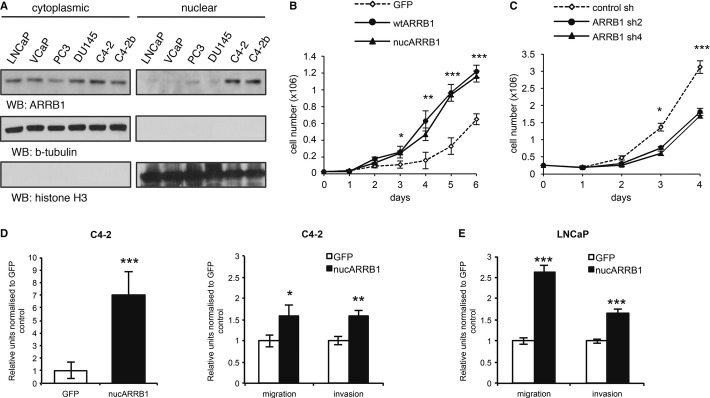
Nuclear ARRB1 levels correlate with the aggressiveness of the cell line Cytoplasmic versus nuclear levels of ARRB1 in a panel of prostate cancer cell lines.GFP control, nucARRB1 and wtARRB1 proliferation.Proliferation of ARRB1 KD C4-2 cells compared to control.Anchorage-independent growth (left) and migration/invasion (right) potential of C4-2 cells expressing GFP control or nucARRB1.Migration/invasion potential of low ARRB1 LNCaP cells expressing GFP control or nucARRB1. Data information: (B and C) *N* = 3, (D and E) *N* = 6, values are mean ± s.e.m., *P <* *0.05, **P <* *0.01, ***P <* *0.001. Source data are available online for this figure.

In a panel of prostate cancer cell lines, we found that the faster growing, more aggressive and highly tumourigenic and metastatic C4-2s and C4-2bs, and to a lesser extend PC3s and DU145s, display higher nuclear levels of ARRB1 compared to LNCaPs and VCaPs (Fig2A and Supplementary Fig S2A). We used C4-2 cells to generate cell lines stably expressing AcGFP-tagged wild-type (wtARRB1) or nuclear (nucARRB1) ARRB1, as well as stable ARRB1 knock-down (KD) cell lines (Supplementary Fig S2B–E). A subcellular fractionation showed that the expressed constructs localise in the expected intracellular compartments (Supplementary Fig S2F and G). NucARRB1 and wtARRB1 C4-2 cells proliferated faster than control cells, whereas ARRB1 KD decreased cell proliferation (Fig2B–D), implying a dependency on adapter expression. As wtARRB1 localises to both cytoplasm and nucleus (Supplementary Fig S2E and F), this suggests that the nuclear fraction of ARRB1 is largely responsible for the observed effect on cellular proliferation.

Previous studies have shown that ARRB1 is required for chemotaxis, suggesting that it may regulate the spread of cancer cells (Ge *et al*, 2004). We found that nucARRB1 expression in C4-2 cells enhanced the transformed phenotype of the cells as indicated by an increase in anchorage-independent growth as well as migratory and invasive potential, whereas ARRB1 KD cells had the opposite effect (Fig2D and Supplementary Fig S2H). Importantly, when expressed in LNCaPs, a line with lower endogenous levels of ARRB1 than C4-2s, nucARRB1 resulted in a stronger relative increase in migratory and invasive potential (Fig2E). Thus, expression levels of nuclear ARRB1 positively correlate with the neoplastic phenotype of the cells.

### Genomic landscape of ARRB1 in prostate cancer cells and human prostate tissue

**Figure 3:**
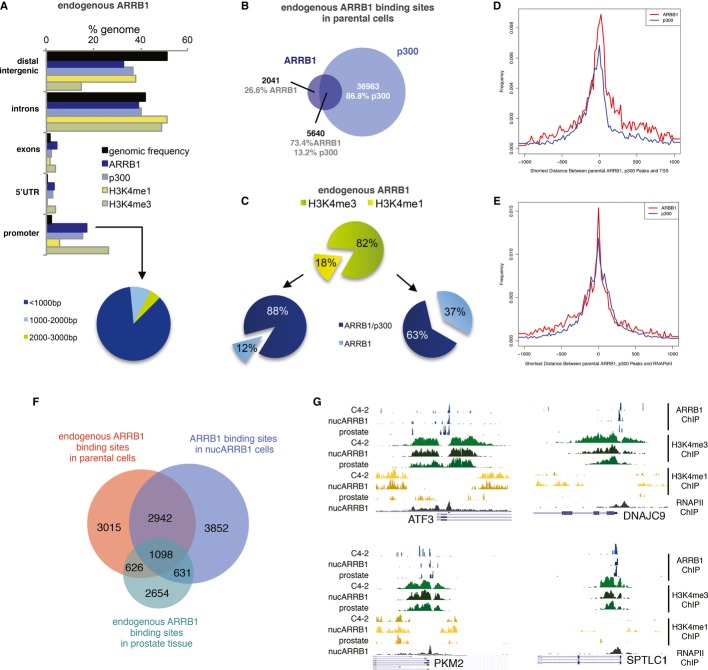
Genomic landscape of endogenous ARRB1 in prostate cancer cells A CEAS-generated genomic distribution of ARRB1, p300, H3K4me1 and H3K4me3 in C4-2s compared to the genomic frequency of the regions considered. The pie chart shows ARRB1-binding sites distribution relative to proximal promoter regions. B Venn diagram showing the overlap (minimum 1 bp) between functional endogenous ARRB1 and p300-binding sites in C4-2 cells. C Distribution of ARRB1-binding sites, either alone or shared with p300, relative to H3K4me1 and H3K4me3 regions in C4-2s. D, E Distance distribution of ARRB1 and p300 peak centres relative to the nearest TSS (D) or RNAPolII site (E). F Venn diagram showing the overlap (minimum 1 bp) between functional endogenous ARRB1 binding sites in human prostate tissue and cell lines. G Integrated Genome Browser view of ChIP-Seq enrichment profiles of ARRB1, H3K4me3, H3K4me1 and RNAPII in parental C4-2, nucARRB1 cell lines and human prostate tissue.

In order to dissect the mechanism behind the tumourigenic role of ARRB1, we generated whole-genome ChIP-seq analysis to identify ARRB1 target genes. As ARRB1 was previously shown to interact with p300 in a complex that regulates transcription (Kang *et al*, 2005), we also generated ChIP-seq data for p300. The C4-2 line was selected as its higher levels of nuclear ARRB1 were better suited for ChIP. We used a previously characterised ARRB antibody for ChIP (Kang *et al*, 2005) together with the Illumina platform for library preparation and sequencing. The MACS algorithm (Zhang *et al*, 2008) was used to identify peaks compared to their matched inputs. A total of 11,129 and 41,727 binding sites for ARRB1 and p300, respectively, were identified (Supplementary Fig S3A). Genomic distribution analysis using CEAS (Ji *et al*, 2006) revealed ARRB1 sites to be enriched at gene-proximal regions with 17.3% of binding sites located within promoters (0–3,000 bp from transcription start sites (TSS)) and 38.9% in intronic regions (Fig3A). The majority (86.1%) of promoter-associated ARRB1 sites were situated within 1,000 bp of TSS (Fig3A). Such a distribution suggests recruitment to *cis*-regulatory elements and supports a transcriptional role for ARRB1 in our system.

To refine our data and focus on functionally active regions of the genome, we generated whole-genome data sets for mono (H3K4me1)- and tri (H3K4me3)-methylated lysine 4 residue of histone H3, histone markers that are associated with actively regulated genes at enhancer and promoter regions, respectively (Barski *et al*, 2007; Heintzman *et al*, 2007, 2009; Wang *et al*, 2008, 2009; Hon *et al*, 2009; Bernstein *et al*, 2013). We identified 129,397 and 29,172 binding sites for H3K4me1 and H3K4me3, respectively (Fig3A and Supplementary Fig S3A). Out of the binding sites initially identified, 7,681 ARRB1 and 39,349 p300 peaks were associated with either H3K4me1 or H3K4me3 marks. These were considered as functional loci and selected for subsequent analysis. Comparison of the ARRB1 and p300 cistromes revealed a partial overlap between ARRB1 and p300 binding sites, suggesting that they act in the same regulatory complex (Fig3B). However, the presence of non-overlapping sites indicates that ARRB1 may also modulate transcription independently of p300.

Of the functional ARRB1 loci, 82% overlapped with H3K4me3 regions compared to 18% with H3K4me1, showing a strong bias for promoter regions (Fig3C). Shared ARRB1/p300 sites were preferentially located at proximal promoters (51.7% at H3K4me3 versus to 15.8% at H3K4me1), whereas a lower proportion of sites bound only by ARRB1 was seen at both promoters and enhancers (30.3% and 2.2% at H3K4me3 and H3K4me1, respectively) (Fig3C). Thus, sites shared by ARRB1 and p300 have a greater affinity for promoter regions, consistent with previous reports of a ARRB1/p300 physical interaction within transcriptional complexes at promoter regions (Kang *et al*, 2005; Dasgupta *et al*, 2011). In addition, ARRB1 was found to be tightly centred on TSS and RNA PolII (RNAPII), which typically binds proximal promoter regions of actively transcribed genes, further emphasising ARRB1's affinity for proximal functional regions and suggesting a major role for this protein in gene expression regulation (Fig3D and E). ChIP-seq on nucARRB1 cell lines confirmed these results (Supplementary Fig S3A–F), indicating that constitutive overexpression of nuclear ARRB1 does not alter its genomic landscape.

Having identified the genomic landscape of ARRB1 in prostate cancer cell lines, we validated our findings *in vivo* by examining its binding to chromatin in human prostate tissue (Supplementary Fig S3A). A high proportion of ARRB1 sites (66.5%) were associated with the functional markers H3K4me1 or H3K4me3. Out of these, 47% overlapped with the sites identified in both parental C4-2 and nucARRB1 cell lines (Fig3F). Comparison of the ARRB1, H3K4me1 and H3K4me3 peaks from ChIP-seq in cell lines and human prostate tissue at several representative loci using the Integrated Genome Browser (IGB) illustrates the consistency between the samples (Fig3G).

### ARRB1 regulates the expression of metabolic genes

**Figure 4:**
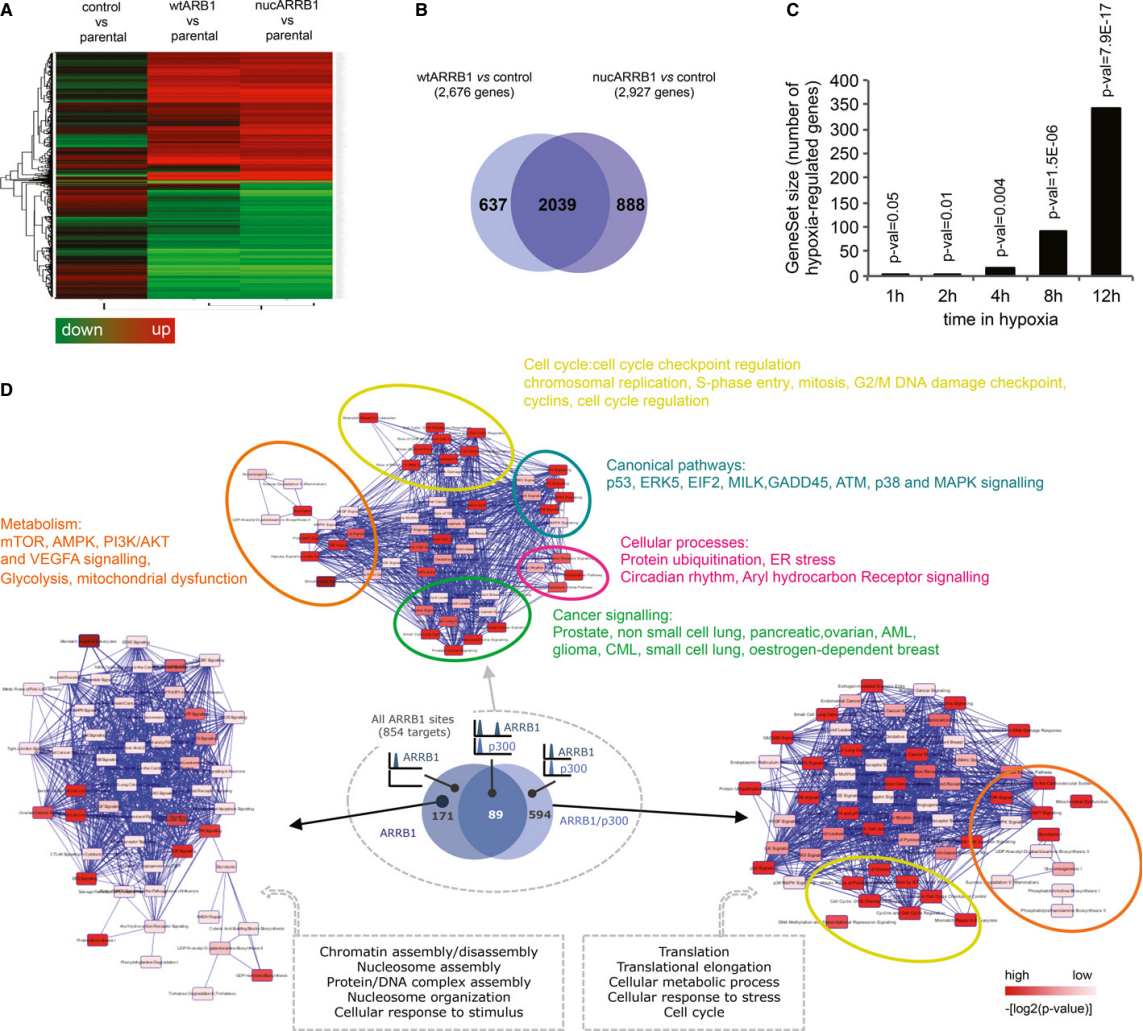
Characterisation of the ARRB1 transcriptome Gene expression heatmap showing ARRB1-regulated genes in control GFP, wtARRB1 or nucARRB1 versus parental C4-2 control.Overlap between DEG in wtARRB1 and nucARRB1.GSEA-enrichment analysis for hypoxia-responsive genes between normoxic nucARRB1 DEG and DU145 prostate cancer cells incubated for 1, 2, 4, 8 and 12 h in hypoxic conditions.Ingenuity Pathway Analysis (IPA) of the 854 direct ARRB1 transcriptional targets. The small Venn diagram cartoon shows the number and overlap of genes in the different categories. IPA analyses of the p300/ARRB1- or ARRB1 alone-regulated genes subgroups are also shown.

To determine the effect of ARRB1 on gene expression in prostate cancer cells, we performed genome-wide expression profiling using Illumina bead arrays. WtARRB1 and nucARRB1 cell lines displayed clearly different clustering and gene expression patterns compared to control cell lines (Fig4A and Supplementary Fig S4A), and a large fraction of differentially expressed genes (DEGs) were common to both wtARRB1 and nucARRB1, suggesting that the nuclear pool of ARRB1 is responsible for many of the changes in gene expression associated with increased levels of ARRB1 (Fig4B and Supplementary Table S1A and B). Real-time PCR validation of the gene expression profiling yielded an experimental false discovery rate of approximately 1.6% (Supplementary Fig S4B).

Functional analysis of the nucARRB1 transcriptome using DAVID gene ontology (GO) analysis revealed an enrichment of genes involved in cellular metabolism and the cell cycle (Supplementary Fig S4C and Supplementary Table S1C). Of note, within the ARRB1-regulated genes, we identified an overlap with the HIF1 transcriptome including known HIF1A targets such as genes involved in angiogenesis (*VEGFA* and *VEGFB*), glycolysis (*ALDOA, ALDOC*, *ENO3*, *PGM1, HK2*), glucose transport (*GLUT12*), mitochondrial function (*MXI1, BNIP3, BNIP3L*), oxygen consumption (*LONP1*), lipid synthesis (*PPARG*) and proliferation (*STC2*). To confirm this in an unbiased manner, we correlated the gene expression profiling data set obtained in nucARRB1 to that of a recent study reporting the hypoxia-induced transcriptional response in DU145, a prostate cancer cell line (Starmans *et al*, 2012). Gene Set Enrichment Analysis revealed a robust correlation between the two data sets as early as two hours incubation in hypoxia, confirming a hypoxic signature in our data set (Fig4C).

qPCR on selected genes in ARRB1 KD versus control or GFP versus nucARRB1, wtARRB1 and Q394L ARRB1, a previously characterised ARRB1 mutation that prevents the translocation of ARRB1 to the nucleus and keeps it solely cytoplasmic, confirmed the effect of nuclear ARRB1 on metabolic gene transcription (Scott *et al*, 2002; Wang *et al*, 2003) (Supplementary Fig S4D and E).

In order to find direct transcriptional targets of ARRB1, we integrated the ChIP data and gene expression profiling obtained in nucARRB1 cells and derived a core set of 854 potential direct transcriptional target genes (Supplementary Fig S4F and G and Supplementary Table S1D). DAVID and IPA GO analyses revealed cellular metabolism and cell cycle amongst the most significant pathway networks associated with ARRB1's direct transcriptional targets (Fig4D, Supplementary Fig S4H and I and Supplementary Table S1E and F). Importantly, targets exclusively associated with ARRB1 sites are closely related to the cell cycle (nucleosome organisation, chromatin and nucleosome assembly and disassembly, protein/DNA complex assembly), whereas the vast majority of targets associated with both ARRB1 and p300 are linked to cellular metabolism and cell cycle (Fig4D).

Functional annotation of the potential direct ARRB1 targets in human tissue also revealed metabolic processes in the most highly enriched subsets (Supplementary Table S1G), indicating that ARRB1 is likely to regulate the same cellular processes in cell lines and tissue and is closely associated with metabolic processes both *in vitro* and *in vivo*.

As nuclear ARRB1 levels are increased in prostate cancer, we hypothesised that gene expression might be dysregulated in a similar way in nucARRB1 and prostate cancer tissue. Five independent clinical gene expression studies showed this to be the case for multiple ARRB1 target genes (Supplementary Fig S4J). These results revealed a conserved gene expression signature dependent of ARRB1 both in cell lines and, more importantly, in human prostate tumours.

### ARRB1 interacts with and regulates the transcriptional activity of HIF1A in a HIF1-dependent fashion

**Figure 5:**
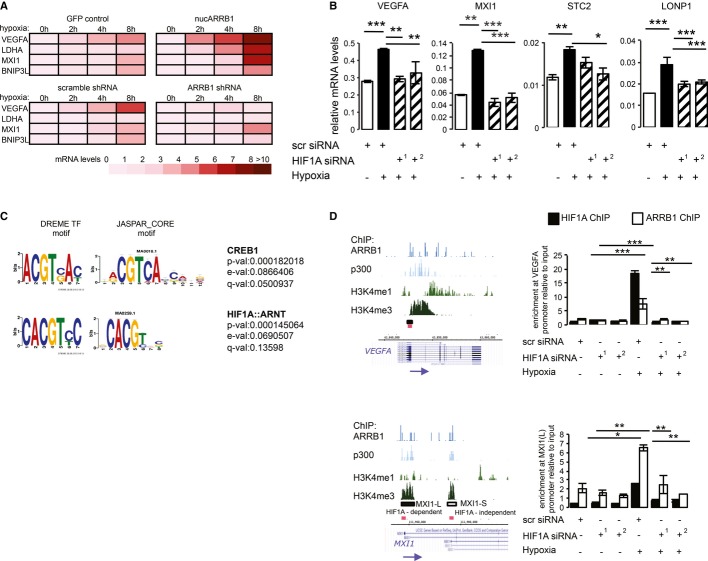
ARRB1 modulates hypoxia-induced HIF1A transcriptional activity GFP control and nucARRB1 (top) or scramble shRNA and ARRB1 shRNA (bottom) cells were incubated in hypoxia (1% O_2_) for 0, 2, 4 or 8 h. Heatmap showing expression levels of HIF1A metabolic target *genes VEGFA, LDHA, MXI1* and *BNIP3L* measured by qRT-PCR.Expression of HIF1A target genes in nucARRB1 cells transiently transfected with scramble (scr) or two different HIF1A siRNAs (siRNA1 and siRNA2) and grown in hypoxia (1% O_2_ for 8 h) 48 h post-transfection.TF motif over-representation using DREME. The ARRB1-associated matched motifs (left) are compared to motifs in the JASPAR_CORE database (right). TF name, *P*-value (probability that the match occurred by random chance according to the null model), *E*-value (expected number of false positives in the matches) and *Q*-value (minimum false discovery rate required to include the match) are shown.HIF1A and ARRB1 recruitment to the chromatin was assessed by ChIP followed by qRT-PCR. NucARRB1 cells transfected with scramble or two different HIF1A siRNAs. Fourty-eight hours after transfections, the cells were incubated in normoxia or hypoxia (1% O_2_ for 12 h). Left: ChIP-seq-enrichment profiles of endogenous ARRB1, p300 and H3K4me1 and H3K4me3 in C4-2s indicating the genomic location of HIF1A-binding sites (black boxes) as reported in Lofstead *et* *al*. The red boxes indicate the genomic region selected for designing the primers used to amplify the immunoprecipitated chromatin. MXI1-L and MXI1-S indicate HIF1A-binding sites regulating the two different MXI1 isoforms. Transcriptional regulation of the MXI1-L-associated isoform has been shown to be HIF1A dependent, whereas that of the MXI1-S-associated isoform is HIF1A independent. Right: ARRB1- and HIF1A-enrichment normalised to input and amount of chromatin at the VEGFA and MXI1-L sites. Data information: For all graphs, *N* = 3, values are mean ± s.e.m. *P < 0.05, **P < 0.01, ***P < 0.001.

As ARRB1 occupies the promoters and modulates the expression of HIF1A targets genes, we tested whether it contributed to the HIF1A-dependent hypoxic transcriptional response. We performed real-time quantitative PCR analysis of mRNA extracted from control and nucARRB1 or ARRB1 KD cells cultured under normoxic or hypoxic conditions (1% O_2_ for 2, 4 or 8 h) to assess the expression of HIF1A targets. Nuclear expression of ARRB1 combined with hypoxia had additive effects on the expression of HIF1A target genes, whereas ARRB1 KD prevented the full induction of most of the targets (Fig5A and Supplementary Fig S5A).

Next, we queried whether HIF1A was required for ARRB1-mediated target gene expression. Hypoxic stimulation of nucARRB1 cells resulted in an increase in target gene expression that was reverted by HIF1A KD, suggesting that nucARRB1's effect is dependent on HIF1A (Fig5B and Supplementary Fig S5B and C). Altogether, these results demonstrate a role for ARRB1 as a regulator of hypoxia-mediated HIF1A transcriptional activity in prostate cancer cells.

To date, ARRB1 has not been reported to bind directly to DNA, but, as a scaffold protein, it is likely to do so via interaction with chromatin-binding proteins, such as TFs. Motif co-enrichment can successfully predict TF associations at the protein level and provide predictions of the composition of transcriptional complexes. Using *de novo* motif discovery and known motifs over-representation on the ARRB1-associated sequences identified in parental and nucARRB1 cells, the HIF1A::ARNT binding motif was identified as the most significant (Fig5C). The CREB motif was also identified in both data sets. This concurs with previous reports of interactions between ARRB1 and these two TFs.

As ARRB1 and HIF1A modulate the expression of a similar set of genes, we tested whether this activity was a result of their physical interaction. Using nuclear extracts from hypoxia-treated ARRB1-expressing cells or GFP control to co-immunoprecipitate ARRB1 and HIF1A using an anti-GFP antibody, we demonstrate that ARRB1 and HIF1A interact in the cell's nuclear compartment but not in the cytoplasmic one (Supplementary Fig S5D and E). An interaction was also detected in co-IPs from nuclear extracts of hypoxia-treated C4-2 cells expressing endogenous levels of ARRB1 (Supplementary Fig S5F). These findings demonstrate that ARRB1 and HIF1A physically interact in the nuclear compartment of prostate cancer cells and, together with the effect on gene expression, suggest that ARRB1 might act as a co-regulator of HIF1A activity. HIF2A is a hypoxia-induced transcriptional regulator closely related to HIF1A that also activates hypoxia response elements (HRE)-dependent gene transcription (Wenger, 2002). However, studies have shown HIF1A and HIF2A to be non-redundant, to activate distinct transcriptional targets and to promote different tumour growth (Kaelin, 2002; Covello *et al*, 2005; Rankin *et al*, 2007). Importantly, we did not detect any interaction between ARRB1 and HIF2A (Supplementary Fig S5F). In addition and consistent with this, HIF2A expression was below detection levels in all prostate cancer cell lines tested under normoxia and only detected in PC3s and DU145s under hypoxic conditions (Supplementary Fig S5G), thus validating the absence of ARRB1/HIF2A interaction and explaining the lack of compensation following HIF1A knock-down in our system.

As ARRB1 itself cannot bind chromatin, we sought to ask whether HIF1A was required for its recruitment to the chromatin. By comparing our data with HIF1A ChIP data from two previous studies (Lofstedt *et al*, 2009; Xia & Kung, 2009; Xia *et al*, 2009), we identified *VEGFA*, *STC2*, *LONP1* and *MXI1* as joint ARRB1/HIF1A transcriptional targets that contain HREs within their promoters are upregulated by nucARRB1 and are induced by hypoxia in a HIF1A-dependent manner (Supplementary Fig S5H). Using ChIP followed by qRT-PCR, we mapped the recruitment of both ARRB1 and HIF1A to these sites within the *VEGFA* and *MXI1* promoters. PCR analysis of the immunoprecipitated chromatin indicated increased occupancy of both HIF1A and ARRB1 at the promoter regions following hypoxic treatment compared to normoxic conditions (Fig5D). HIF1A KD resulted in the loss of both HIF1A and ARRB1 recruitment, suggesting that, upon hypoxia, HIF1A recruits ARRB1 to the regulatory regions of its transcriptional targets. No enrichment was observed at a distal HIF1A-independent control region (Supplementary Fig S5I). Together with our gene expression analysis, this suggests that the physical interaction between ARRB1 and HIF1A at the regulatory regions of HIF1A target genes is required for their expression. Thus, ARRB1 acts a co-regulator of HIF1A activity in prostate cancer cells.

### ARRB1 induces the pseudohypoxic stabilisation of HIF1A by controlling the expression of TCA cycle enzymes FH and SDH

**Figure 6:**
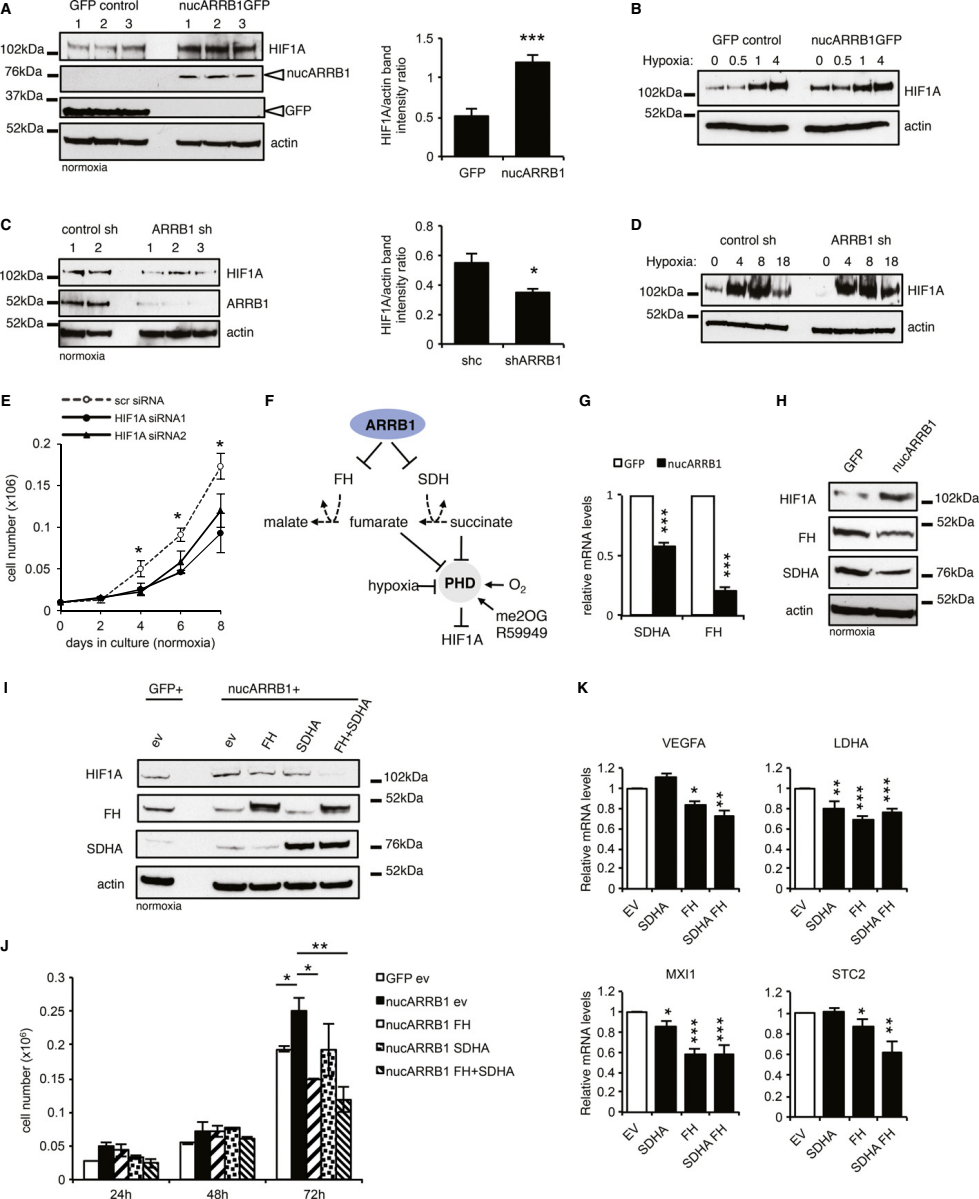
Nuclear ARRB1 induces pseudohypoxia Left: immunoblot showing increased levels of HIF1A in three nucARRB1 cell lines compared to GFP control. Right: ImageJ quantification of the bands on the left.Immunoblot of GFP control and nucARRB1 incubated under hypoxic conditions (1% O_2_) for increasing periods of time.Left: Immunoblot of two control shRNA and three ARRB1 shRNA cell lines showing a reduction in HIF1A levels in ARRB1 KD cell lines. ARRB1 and β-actin loading control are also shown. Right: ImageJ quantification of the bands on the left.HIF1A stabilisation in control and ARRB1 knock-down cell lines incubated in hypoxia (1% O_2_) for increasing periods of time.NucARRB1 cells were transfected with scramble or two different HIF1A siRNAs. Cell numbers were determined after 2, 4, 6 and 8 days.Schematic diagram of the regulation of HIF1A by various stimuli including oxygen tension, oncometabolites succinate and fumarate and PHD activators me2OG and R59949. ARRB1 may regulate HIF1A stability through modulation of *FH* and *SDH* expression.*SDHA* and *FH* mRNA expression levels of in nucARRB1 cells relative to control.Normoxic levels of HIF1A, FH and SDHA in GFP and nucARRB1 cell extracts.HIF1A, FH and SDHA levels in nucARRB1 cells transiently transfected with empty vector (EV), FH, SDHA or both FH and SDHA expression vectors. EV-transfected GFP cells were used a baseline control. Extracts were harvested 48 h post-transfection.NucARRB1 cell numbers described in G above were determined 24, 48 and 72 h after transfection.Relative mRNA expression levels of HIF1A metabolic targets from cells described in (G) were measured by qRT-PCR 48 h after transfection. Data information: For all graphs, *N* = 3, values are mean ± s.e.m., *P <* *0.05, **P <* *0.01, ***P <* *0.001. Source data are available online for this figure.

Given the hypoxic signature observed in nucARRB1 cells under normoxic conditions, we investigated whether ARRB1 is able to modulate HIF1A stability. Extracts from normoxic nucARRB1 cells showed increased levels of HIF1A relative to control (Fig6A). When these cells were cultured under 1% O_2_, HIF1A was stabilised earlier in nucARRB1 compared to control cells (Fig6B). Conversely, ARRB1 KD resulted in lower levels of HIF1A protein, although this did not prevent hypoxic stabilisation of HIF1A (Fig6C and D). HIF1A expression is critical for cancer cell growth, and its knock-down impairs proliferation (Semenza, 2000; Seagroves *et al*, 2001). NucARRB1 proliferate faster than control cells, a phenotype that is dependent on HIF1A expression as its knock-down results in slower growth (Fig6E). Stabilisation of HIF under normoxia via competitive inhibition of PHDs has been previously demonstrated to occur from the accumulation of TCA cycle intermediates succinate and fumarate as a result of the loss of *succinate dehydrogenase* (*SDH*) or *fumarate hydratase* (*FH)* expression (Fig6F). Remarkably, our gene expression profiling showed a downregulation of *SDHA* and *FH* in nucARBBB1 cells, a result that was confirmed by qRT-PCR (Fig6G). NucARRB1 cells also showed reduced FH protein levels compared to control (Fig6H, lanes 1 and 2 in Fig6I). We thus assessed whether the downregulation of *SDHA* and *FH* by nucARBB1 is instrumental for subsequent HIF1A stabilisation under normoxia by overexpressing *SDHA* and *FH* in nucARRB1 cells (Fig6I and Supplementary Fig S6A and B). This resulted in a reduction of HIF1A protein levels accompanied by a reduction in proliferation rate (Fig6I and J) and downregulation of the metabolic HIF1A targets genes (Fig6K). Expression of both *FH* and *SDHA* had an additive effect on HIF1A stability and cell proliferation.

### ARRB1 expression levels in prostate cancer cells determine their sensitivity to HIF1A signalling inhibition

We have shown earlier that faster growing, tumourigenic and metastatic prostate cancer cell lines express higher nuclear levels of ARRB1 (Fig2A and Supplementary Fig S2A). Crucially, these cells also display higher HIF1A levels under normoxic conditions, and, generally, cells with low levels of nuclear ARRB1 also show lower HIF1A levels under normoxia (Supplementary Fig S6C and D). Compared to the low ARRB1/HIF1A-expressing LNCaP line, the high ARRB1/HIF1A metastatic derivative C4-2 line also shows lower FH expression levels (Supplementary Fig S6E), suggesting a likely similar HIF1A stabilisation mechanism to that observed in nucARRB1 cells. Using these two lines, we investigated the effect of pharmacological inhibition of HIF1A signalling. Dimethyl-2-oxoglutarate (me2OG) and R59949 both have been shown to reactivate PHD and result in inhibition of HIF1A signalling (Temes *et al*, 2005; MacKenzie *et al*, 2007). Incubation with these compounds resulted in lowering the levels of HIF1A in C4-2 cells (Supplementary Fig S6F). This effect was accompanied by a stronger relative cell growth reduction in C4-2 compared to LNCaPs, indicating that PHD reactivation is more effective in reducing proliferation of cells with higher basal levels of HIF1A and nuclear ARRB1, such as C4-2s (Supplementary Fig S6G).

### ARRB1 reprograms cell metabolism

**Figure 7:**
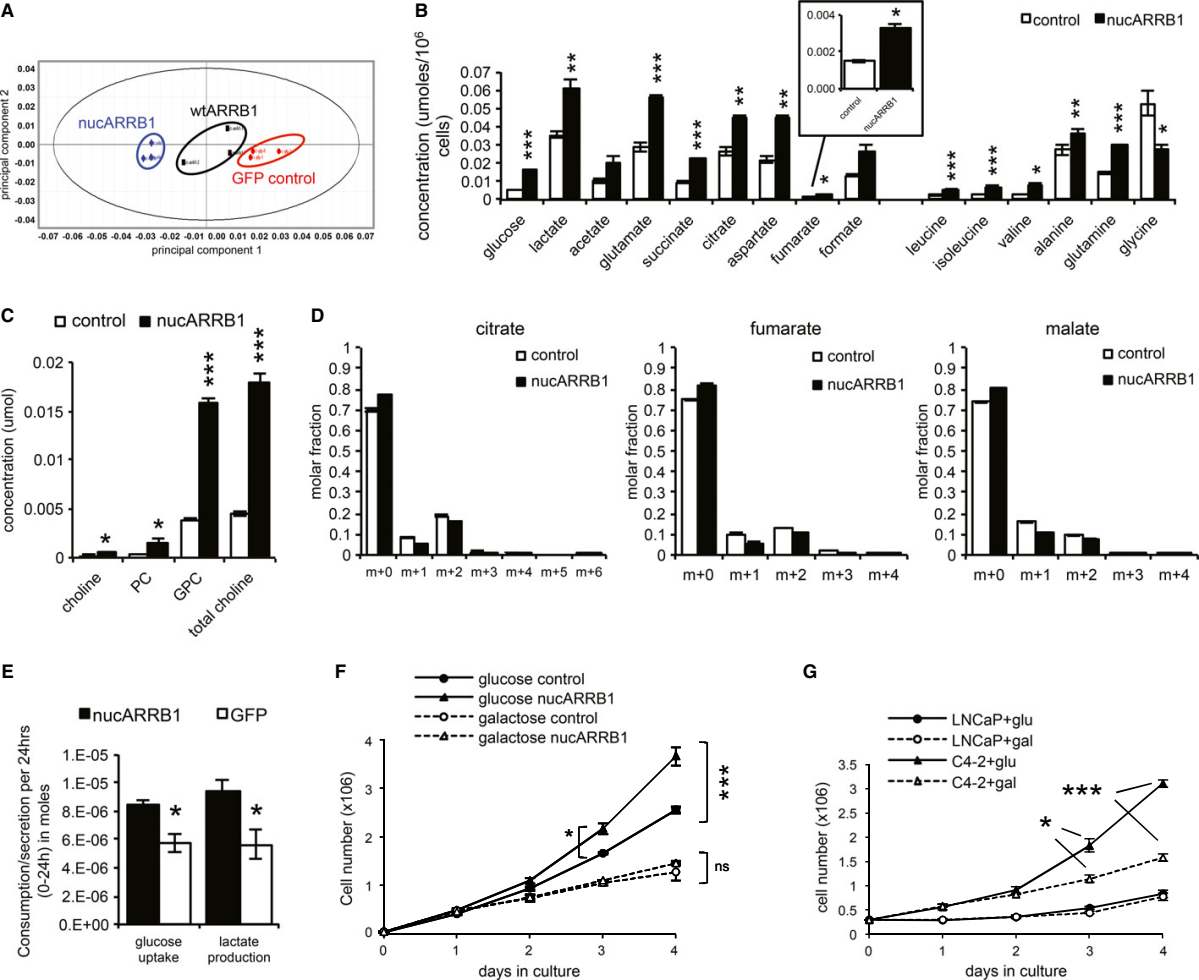
ARRB1 regulates metabolism in prostate cancer cells Principal Component Analysis of ^1^H NMR proton nuclear magnetic resonance measurements of cellular metabolites in control, wtARRB1 and nucARRB1 cells.^1^H NMR proton nuclear magnetic resonance measurements of intracellular glucose and lactate, amino acids and TCA intermediate in GFP control and nucARRB1 cells.Kennedy pathway phospholipid intermediate metabolites choline, phosophocholine (PC), glycerophosphocholine (GPC) and total choline in GFP control and nucARRB1 cell lysates.Measurment of glucose flux to fumarate, malate and citrate using 1,2-^13^C-glucose and GC/MS in control and nucARRB1 cells.Glucose uptake and lactate secretion in GFP control and nucARRB1 cells.Growth plots of GFP control and nucARRB1 cells cultured in media supplemented with either glucose or galactose.Proliferation plots of LNCaP and C4-2 cells grown in media supplemented with either glucose or galactose. Data information: For (B–E), values are mean ± s.e.m. of five replicates. For (F–G), *N* = 3, values are mean ± s.e.m. *P <* *0.05, **P <* *0.01, ***P <* *0.001.

Given the induction of a pseudohypoxic response that can be reversed by re-expressing *SDHA* and *FH*, we asked whether ARRB1 might have broader effects on cancer cell metabolism. In particular, this raises the possibility that accumulation of succinate/fumarate could be responsible for the pseudohypoxic activation of HIF seen in nucARRB1 cells. To investigate this possibility, we examined global cell metabolism using proton nuclear magnetic resonance (^1^H NMR) in nucARRB1 cells. Spectra analysis of control, wtARRB1 and nucARRB1 cell extracts revealed that these cells cluster into three different metabolic groups (Fig7A). We found nucARRB1 to induce significant changes in the concentrations of metabolites involved in glucose and amino acid metabolism as well as TCA cycle metabolite concentrations (Fig7B). Importantly, and consistent with our hypothesis, both succinate and fumarate concentrations were higher in nucARRB1 cells. Intracellular glucose and lactate were also significantly higher in nucARRB1 compared to control cells (Fig7B), suggesting an increase in glucose import and a stimulation of aerobic glycolysis; in contrast, these were decreased in ARRB1 KD (Supplementary Fig S7A). We also noticed a stimulation of anabolic synthesis as shown by increased cellular concentrations of amino acids in nucARRB1 cells (Fig7B). Overexpression of nuclear ARRB1 also resulted in significantly higher levels of total choline content, as well as phosphocholine (PC) and glycerophosphocholine (GPC), consistent with a more malignant, faster proliferating phenotype, whereas ARRB1 KD had the opposite effect (Fig7C and Supplementary Fig S7B).

It has been demonstrated that HIF-mediated metabolic reprogramming would shunt glucose-derived carbons out of the mitochondria via the inhibition of pyruvate dehydrogenase (PDH) and therefore reduce the flux of glucose-derived carbon to the TCA cycle. As we observed an increase rather than a decrease in TCA cycle metabolites, we investigated the effects of nucARRB1 overexpression on carbon supply to the TCA cycle. To this aim, nucARRB1 cells were cultured in medium supplemented with ^13^C-labelled glucose, and isotopologue analysis of fumarate, citrate and malate was performed. The majority of these metabolites were unlabelled (m + 0), indicating that glucose is not the major source of carbon for the TCA cycle in these cells (Fig7D). Of note, upon the expression of nucARBB1, a small but significant decrease in the m + 2 (glucose-derived) and an increase in the unlabelled isotopologues were observed (Fig7D), suggesting that nucARRB1 promotes a diversion of glucose-derived carbons out of the mitochondria and, more importantly, that other carbon sources might be responsible for the accumulation of these metabolites in nucARRB1 cells.

To confirm that the metabolic pattern seen in nucARRB1 cells reflects an increase in glycolysis, we measured glucose uptake and lactate production. NucARRB1 cells consumed more glucose and secreted more lactate into the medium than the control cells, suggesting that increased levels of nuclear ARRB1 alter cellular metabolism to promote aerobic glycolysis (Fig7E). We therefore tested the dependency of nucARRB1 cells on aerobic metabolism of glucose by assessing their growth in media supplemented with galactose instead of glucose. Under such conditions, the reduction in glycolytic flux would force the cells to rely on oxidative phosphorylation to proliferate. While nucARRB1 cells grew significantly faster than control cells in glucose-containing medium, both cell lines grew at the same slower rate in galactose-supplemented medium, implying that nucARRB1 cells rely on glucose catabolism to sustain their increased proliferation rate (Fig7F). Similarly, whereas LNCaPs grew equally well in glucose- or galactose-supplemented medium, C4-2s’ growth rate was significantly slowed down in galactose-supplemented medium, indicating their reliance on glycolysis (Fig7G).

Thus, our metabolomic analysis indicates that overexpression of nuclear ARRB1 in prostate cancer cells promotes profound metabolic changes that would fuel the increased energy demands of growth and proliferation of cancer cells.

## Discussion

Herein, we present the first whole-genome description of the transcriptional networks and associated pathways controlled by an endocytic adaptor, ARRB1, in prostate cancer cells. We show that ARRB1 physically occupies the promoter regions and modulates the expression of genes involved in cellular metabolism. We report a nuclear physical interaction between ARRB1 and HIF1A in prostate cancer cells and show that it occurs at the chromatin level, where recruitment of ARRB1 to HREs at functional promoters of HIF1A targets upon hypoxia is HIF1A dependent. Consistent with a role as a HIF1A co-factor, we show that ARRB1 facilitates HIF1A-regulated gene expression and that this effect is further enhanced under hypoxic conditions. In addition, lowering ARRB1 levels, although significantly reducing transcriptional activity, does not abolish the HIF1A-mediated response that exists at basal levels in C4-2s even under normoxic conditions, thereby strengthening the hypothesis that ARRB1 has a role as a facilitator of HIF1A transcriptional activity. A recent study describing a role for ARRB1 in HIF1A-dependent *VEGFA* expression in breast cancer cells supports our findings (Shenoy *et al*, 2004).

The transcriptional activity of ARRB1 on HIF1A target gene expression reported here was observed in normoxia as well as in hypoxia, indicating that ARRB1 can regulate HIF1A stability under normoxic conditions. Moreover, the ARRB1-mediated metabolic alterations reported here also occur in normoxia. HIF1 activity can be stimulated in well-oxygenated environments that have disrupted oxygen-sensing controls (Kaelin & Ratcliffe, 2008; Aragones *et al*, 2009), but HIF1A stabilisation can also occur under normoxic conditions as a consequence of mutations in metabolic genes. For example, rising succinate and fumarate concentrations resulting from mutations in *SDH* subunits and *FH* have been shown to inhibit PHD activity, a process called pseudohypoxia (Frezza *et al*, 2011; Cardaci & Ciriolo, 2012). The role of pseudohypoxia in tumour formation has been well documented (Raimundo *et al*, 2011). Somatic mutations in *SDH* and loss-of-function germline mutations in *FH* have been associated with many cancers (Tomlinson *et al*, 2002; Lehtonen *et al*, 2006; Korpershoek *et al*, 2011). Tumours derived from *SDH* or *FH* mutations are characterised by a strong hypoxic signature and are significantly more vascularised (Dahia *et al*, 2005; Vanharanta *et al*, 2006; Favier *et al*, 2012). In addition to genetic defects resulting in loss-of-function in SDH or FH subunits, pseudohypoxia has also been showed to arise as a consequence of transcriptional regulation of SDHA (Piantadosi & Suliman, 2008). We show here that high levels of nuclear ARRB1 in prostate cancer cells result in downregulation of *FH* and *SDHA* expression, although this effect is likely to be an indirect consequence as our genomics analysis did not identify any ARRB1-binding sites in the promoter regions of these two genes. A reduction in FH and SDH complex activities should result in a significant decrease in oxidative phosphorylation necessitating a reliance on glycolysis followed by conversion of pyruvate to lactate to provide adequate energy, an effect confirmed by the metabolic profile seen in nucARRB1 cells.

First described by Otto Warburg in the 1920s, the ‘Warburg effect’ postulates that, in contrast to normal cells, which mainly use oxidative phosphorylation for growth and survival, tumour cells rely on enhanced aerobic glycolysis as their major source of energy to fuel cellular proliferation. Since then, this shift in metabolism has emerged as a hallmark of many cancers (Semenza, 2007a; Dang, 2012; Locasale, 2012). We show here that high levels of nuclear ARRB1 result in an increase in glucose intake and lactate secretion, consistent with the increase in glycolytic gene expression seen in these cells (Supplementary Table S1A and B). In addition, ARRB1 might contribute to mitochondrial dysfunction via regulation of other HIF1A target genes, such as *MXI1*, *BNIP3* and *BNIP3L* (Supplementary Table S1A and B).

Increasing evidence suggests that enhanced glucose catabolism and reduced mitochondrial function in tumour cells result in an increase in anabolic substrates (Tong *et al*, 2009). Consistent with this, we observed an increase in amino acids in nucARRB1-overexpressing cells. Cells with high nuclear ARRB1 levels also display increased levels of the choline phospholipid metabolites PC and GPC. A study in a human prostate cancer model has shown that hypoxic activation of HIF1A signalling results in a similar effect on these metabolites (Glunde *et al*, 2008). Moreover, increased phospholipid turnover has been reported in several cancers, including prostate, breast, colon and brain and is often associated with malignant transformation, invasion and metastasis (Ackerstaff *et al*, 2001; Teahan *et al*, 2011).

Our study is the first to demonstrate that an endocytic adaptor contributes to the Warburg effect by modulating the expression levels of metabolic enzymes and the concentrations of cellular metabolites. By increasing glycolytic activity and decreasing mitochondrial function, an adaptive mechanism used by cancer cells to fuel their growth, ARRB1 would therefore act as a tumour promoter.

**Figure 8:**
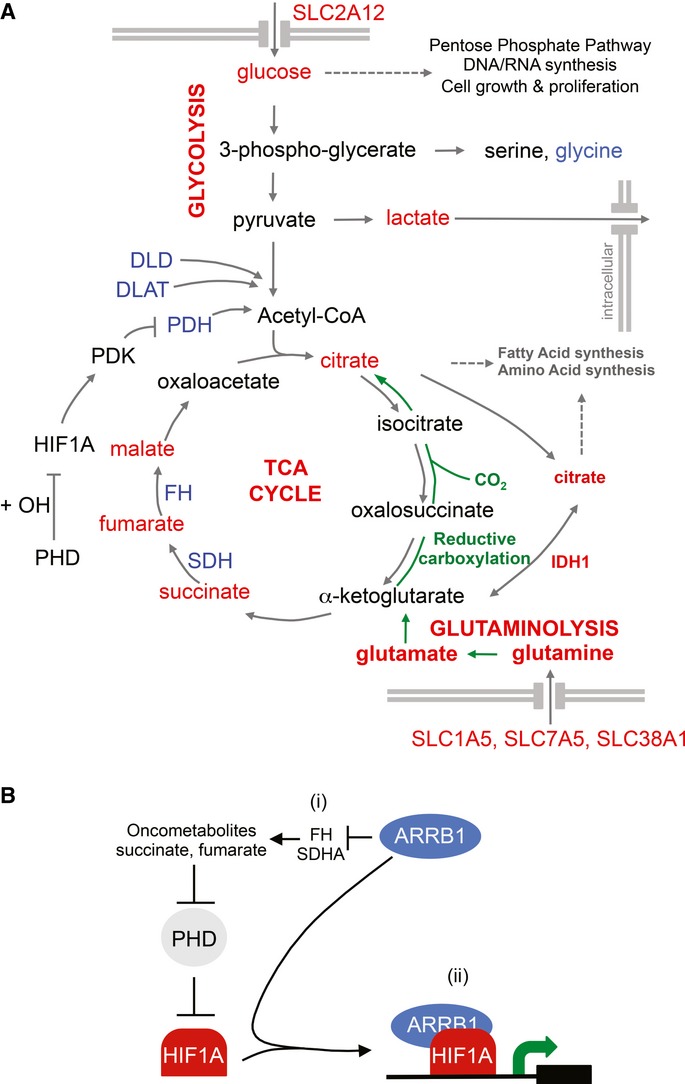
ARRB1-mediated effect on cellular metabolism and stabilisation/activation of HIF1A signalling Schematic diagram illustrating metabolites of the glycolytic pathway and the TCA cycle that are altered by ARRB1. In red are metabolites or genes showing increased concentration or expression, respectively, in nucARRB1; in blue are those with lower concentration or expression, respectively. Reductive carboxylation pathway of glutamine is indicated in green.Modulation of HIF1A signalling by ARRB1 occurs at two levels: (i) ARRB1 indirectly alters expression levels of metabolic enzymes FH and SDHA, resulting in accumulation of oncometabolites fumarate and succinate and pseudohypoxic stabilisation of HIF1A, and (ii) ARRB1 acts as a co-factor of HIF1A to enhance its transcriptional activity both in normoxic and hypoxic conditions.

Overexpression of nuclear ARRB1 also resulted in downregulation of *PDHA1* (0.5-fold)*, DLAT* (0.6-fold) and *DLD* (0.8-fold), three enzymes that are involved in the conversion of pyruvate into acetyl-CoA (Fig8A and Supplementary Table S1B). We speculate that this would result in weakening the primary link between glycolysis and the TCA cycle, thus diverting glucose carbons into the production of lactate rather than into the Krebs cycle. Added to the HIF1A stabilisation/activation reported here, this could strengthen further the effect of ARRB1 on cell metabolism.

Glutamine conversion to glutamate and subsequently to α-ketoglutarate by the TCA cycle is essential for tumour cell growth and proliferation. Two distinct pathways can contribute to glutamine-derived lipogenesis: α-ketoglutarate can be oxidatively metabolised in the TCA cycle to generate pyruvate from malate by glutaminolysis (DeBerardinis *et al*, 2007), or it can be reductively carboxylated to generate citrate (Des Rosiers *et al*, 1995; Yoo *et al*, 2008). In hypoxic conditions, *de novo* lipogenesis relies almost exclusively on the reductive carboxylation of α-ketoglutarate. Recent data provide evidence that the reductive pathway involves IDH1-mediated catalysis in the cytoplasm and plays an important role in cell proliferation at physiological oxygen levels (Metallo *et al*, 2012). Interestingly, in nucARRB1 cells, we observed an increase in *IDH1* expression (2.5-fold) (Fig8A and Supplementary Table S1B) indicating a possible increase in glutamine reduction. By doing so, ARRB1, through HIF1A stabilisation/activation (and through downregulation of *PDHA1*, *DLAT* and *DLD*), would not only favour glycolysis in order to produce the glycolytic intermediates required for nucleotide and phospholipid synthesis (via the pentose phosphate pathway) and the energy necessary for survival under hypoxic conditions but also contribute to the generation of ATP, NADPH, ROS, amino acids, nucleotides and lipids. This hypothesis is supported by our tracer experiment using ^13^C-labelled glucose showing decreased glucose incorporation into TCA metabolites in nucARRB1 cells compared to control cells, suggesting that these metabolites are generated from a carbon source other than glucose, possibly from glutamine via anaplerotic flux. This would also fit with a reduced mitochondrial function as suggested by the upregulation of *MXI1*, *BNIP3* and *BNIP3L* in nucARRB1 cells.

In summary, our observations show that, through HIF1A protein stabilisation and transcriptional activation (Fig8B), nuclear ARRB1 induces metabolic re-programming of prostate cancer cells which drives the expression of tumour-specific phenotypes, such as anchorage-independent growth, migration, invasion and proliferation, and confers on the cells a selective growth advantage that may promote tumour progression. Our findings indicate that the regulation of tumour cell metabolism by ARRB1 could provide an important area for cancer diagnosis and that preventing its nuclear import could be exploited as a therapeutic tool in limiting prostate cancer progression and metastasis.

## Materials and Methods

### Cell culture

All prostate cancer cell lines and 786-O cells were grown in RPMI (Gibco 21875-034) supplemented with 10% foetal bovine serum (Gibco 10270-106). Stable cell lines were generated by nucleofection (Lonza) of the AcGFP fusion ARRB1 or ARRB1 shRNA constructs in C4-2 followed by selection with 0.3 mg/ml final concentration of Geneticin G418 sulphate (Gibco 10131-019) or 10 mg/ml puromycin (Sigma P9620-10ML), respectively, added to the culture medium. Dimethyl-2-oxoglutarate (359631-5G) and R59949 (D5794-5MG) were obtained from Sigma.

### Antibodies used for ChIP

ARRB1 (A1CT, a gift from Professor R. Lefkowitz), p300 Santa Cruz rabbit polyclonal (sc-585), H3K4me1 Diagenode rabbit polyclonal (pAb-037-050), H3K4me3 rabbit polyclonal (pAb-003-050) and HIF1A Novus Biologicals rabbit polyclonal (NB100-134SS).

### Plasmids

A humanised sequence of ARRB1 tagged with 3XNLS sequences was obtained from Sloning BioTechnology and supplied in a pPCRScript vector. nucARRB1 was generated by cloning the (XhoI-EcoRV) fragment into pAcGFP-C1 digested with XhoI and EcoRV to generate pAcGFP-ARRB1-3NLS. wtARRB1 was generated by digesting pAcGFP-ARRB1-3NLS with NheI and cloning the (NheI-NheI) fragment into pAcGFP-C1 (Clontech) digested with NheI and XbaI. This resulted in fusion of the NheI and XbaI sites leading to their deletion. This generated an intermediate construct containing an extra 5 amino acid (AA) tail (LARAR) before the stop codon directly followed by a BclI site. To delete these 5 AA, the intermediate construct was digested with BamHI and BclI, and two 5′-PO4 complementary primers replacing the ARRB1-coding sequence downstream of the BamHI site with the correct sequence including the stop codon (directly followed by a newly introduced NheI site) were hybridised and ligated in BamHI-BclI. pAcGFP-C1 was used as the control vector. FH and SDHA expression plasmids as well as ARRB1 shRNAs were obtained from Origene.

### Immunoblotting and immunofluorescence

Rabbit polyclonal antibody against ARRB (A1CT) was kindly provided by Professor Robert Lefkowitz, Duke University. The following antibodies were used: GFP (AbCam ab6556 and ab291), beta-actin (AbCam ab6276-100), HIF1A (Novus NB100-134SS, AbCam ab2185 and BD Transduction 610958), HIF2A (AbCam ab157249 and ab199), LSD1 (Cell Signaling 2139), FH (AbCam ab113963) and SDHA (AbCam ab137040). The secondary antibodies used for Western blotting were horseradish peroxidase (HRP)-conjugated antibody against mouse immunoglobulin G (IgG) and HRP-conjugated antibody against rabbit IgG from Dako. For immunofluorescence, the secondary antibodies were Alexafluor A488-conjugated antibody against mouse immunoglobulin G (IgG) from Molecular Probes. DAPI was used to stain DNA (nucleus).

### Immunohistochemistry

Paraffin-embedded sections were dewaxed and rehydrated (xylene1 5 min, xylene2 5 min, abs1 5 min, abs2 5 min, 90% etoh 5 min, 80% etoh 5 min, dH_2_O 5 min, PBS 5 min). Antigen retrieval was performed by microwaving the slides in Tris/EDTA buffer (pH 9.0) for 15 min. The slides were cooled down for 20 min and incubated with 1% normal donkey serum for 1 h before incubation with the primary antibody (ARRB1: AbCam ab32099) at a 1:100 dilution for 1 h at room temperature. The slides were then incubated with a 1:200 dilution of biotinylated IgG secondary antibody (Biotin-SP-AffiniPure Donkey Anti-Rabbit IgG (H + L)—Jackson ImmunoResearch Laboratories 711-065-152) for 1 h followed by a streptavidin–biotin–peroxidase detection system (Vectastain Elite ABC Kit). The slides were then visualised using 3,3′-diaminobenzadine (Vector laboratories, SK-4100) and counterstained with haematoxylin (Vector Laboratories H-3404). The stained slides were assessed by a pathologist and scored for ARRB1 intensity and sub-cellular localisation.

### Colony formation, migration and invasion assays

CytoSelect™ 96-Well Cell Transformation Assay, Cell Migration and Invasion Assay kits from Cell Biolabs Inc. were used for assessing anchorage-independent growth/soft agar colony formation, migration and invasion following the manufacturer's instructions. Values were normalised to control mean value (set at 1), and the mean values of the experimental samples were plotted relative to control.

### Tissue microarrays (TMAs) and patient cohorts

Cambridge TMA – Prostate tissue from radical prostatectomies performed at Addenbrookes Hospital, Cambridge, UK, between 2001 and 2005 was used to make TMAs using duplicate 0.6-mm cores taken from paraffin-embedded tissue and a Beecher Manual TMA Arrayer. In total, tissue from 104 different patients was used to generate the TMA. Regions of non-neoplastic prostate and malignancy were identified by a specialist uro-pathologist for each patient. Multiple cores from each patient were used. Pathological stage and Gleason grade were confirmed by a specialist uro-pathologist prior to scoring any IHC staining.

### Chromatin immunoprecipitation

Chromatin immunoprecipitation (ChIP) was performed as previously described (Schmidt *et al*, 2009). Two 15-cm plates of cells were used for each ChIP. Cells were cultured in RPMI media supplemented with 10% FBS. DNA–protein interactions were cross-linked using 1% formaldehyde for 10 min at room temperature, before quenching with a final concentration of 125 mM glycine. Cells were harvested by scraping and washed twice with 10 ml 1× PBS. Nuclear lysates were isolated by incubating cells for 10 min at 4°C in 10 ml of LB1 (50 mM Hepes-KOH, 140 mM NaCl, 1 mM EDTA, 10% glycerol, 0.5% Igepal, 0.25% Triton X-100), pelleting nuclei at 1300 g for 5 min at 4°C, washing nuclei in 10 ml LB2 (10 mM Tris-HCl pH8, 200 mM NaCl, 1 mM EDTA, 0.5 mM EGTA) at 4°C for 5 min, pelleting nuclei as before and adding 1 ml LB3 (10 mM Tris-HCl pH 8, 100 mM NaCl, 1 mM EDTA, 0.5 mM EGTA, 0.1% sodium deoxycholate, 0.5% N-lauroylsarcosine). Nuclear lysates were divided into four 250-ml fractions, sonicated for 15 min (30 s on, 30 s rest) at maximum power in a Bioruptor sonication waterbath (Diagenode), recombined (total volume 1 ml), 100 μl of 10% Triton X-100 was added and insoluble debris was removed by centrifugation at 20,000 *g* for 10 min at 4°C. Supernatants were diluted with 2 ml of LB3 and 200 ml 10% Triton X-100, 50 μl was taken as total input control and the remainder was used for ChIP. For each ChIP reaction, 75 μl protein-A and 75 ml protein-G magnetic beads (Dynal, Invitrogen) were washed three times with 0.5% BSA in 1× PBS, before incubation overnight with 10 μg of specific antibody overnight at 4°C with gentle agitation. Antibody–bead complexes were washed three times in 1 ml 0.5% BSA 1× PBS, resuspended in 100 ml of the same buffer, combined with the pre-cleared nuclear lysates and incubated overnight at 4°C with gentle agitation. The following day bead–antibody–protein–DNA complexes were washed five times in RIPA buffer (50 mM Hepes-KOH pH 7.6, 500 mM LiCl, 1 mM EDTA, 1% Igepal, 0.7% sodium deoxycholate), once with TE plus 50 mM NaCl at 4°C and eluted in 200 ml elution buffer (50 mM Tris-HCl pH 8, 10 mM EDTA, 1% SDS) for 15 min at 65°C with vortexing. Cross-links were reversed overnight at 65°C. RNA and proteins were degraded by adding 200 ml of TE and 8 μg of DNase-free RNase A (Ambion), incubation for 30 min at 37°C, followed by addition of 80 μg proteinase K (Invitrogen) and incubation at 55°C for 1 h. Genomic DNA was isolated using phenol/chloroform/isopropanol (25:24:1, Invitrogen), back-extracted with 200 ml of TE, precipitated with isopropanol, washed with 75% ethanol, air-dried and resuspended in 60 ml 10 mM Tris-HCl pH 8. ChIP enrichment was tested by real-time PCR using 6 ml of DNA, and the remainder was used for single-end Solexa library preparation.

### ChIP-seq Solexa library preparation and analysis

Single-end Solexa sequencing libraries were prepared as previously described (Schmidt *et al*, 2009). Briefly, 54 μl of ChIP DNA or 50 ng of total input control DNA were subjected to end repair using T4 DNA polymerase, Klenow DNA polymerase and T4 polynucleotide kinase, before purification using the DNA Clean and Concentrator-5 kit (Zymo Research). Adenine overhangs were added using Klenow 5′-3′ exo-minus, Illumina Solexa sequencing adapters were ligated using T4 DNA ligase and amplified with 18 PCR cycles using Phusion DNA polymerase (Finnzymes) and Illumina Solexa sequencing primers 1.1 and 2.1. Libraries were size selected by electrophoresis, excising the SYBR-safe, DNA smear between 200–300 bp on a Dark Reader non-UV transilluminator, purified using a Qiagen Gel Extraction MiniElute Kit, quantified using an Agilent Bioanalyser, 36 bp sequence reads were generated using a Illumina (Solexa) Genome Analyzer II and these reads were mapped back to the reference human genome before peak calling.

### GSEA analysis—hypoxic signature

The hypoxia data set, GSE41491 (Starmans *et al*, 2012), was downloaded from the Gene Expression Omnibus database. Gene lists of hypoxia-related genes were created from comparative analyses of hypoxia treated (time-points 1, 2, 4, 8 or 12 h) versus untreated (time 0 h) using limma, a R implemented package available from Bioconductor. The *P*-values were corrected for multiple testing using the Benjamini & Hochberg (1995) method. To test for enrichment in hypoxia-related gene sets, Gene Set Enrichment Analysis was performed on a ranked list of *t*-statistic values using the gene SetTest function of the limma package.

### Metabolomic profiling

^1^H NMR spectroscopy data were acquired on a 600 MHz Bruker Avance NMR spectrometer. Metabolite concentrations were normalised to the number of cells. C4-2 cells were grown in RPMI media supplemented with 10% FBS. Cells were harvested (control on day zero) on day 2. Metabolites from the cells were extracted and their concentrations were estimated using HR-NMR spectral analysis by following our protocol (Madhu *et al*, 2006) which will be described briefly in the following. Media samples were collected from the cell culture dish, and 600 μl of the media sample was used for NMR analysis. After removing the media from the cell culture dish, cells were washed twice with sterile 3 ml physiological saline. 2 ml ice cold 6% PCA was added and cells were scrapped into a centrifuge tube. Scrapped cells were centrifuged at 1,000 rpm for 10 min at 4°C. Supernatant was taken and neutralised to pH 7 with KOH and PCA. After neutralisation and lyophilisation, these intracellular metabolite extract samples were re-suspended in 1 ml of D_2_O for ^1^H NMR analysis. 600 μl of the sample was taken in a 5-mm standard Wilmad NMR tube. Ten μl of 10 mM TSP was added as external standard. ^1^H NMR spectroscopy data were acquired on a 600 MHz Bruker Avance NMR spectrometer. We have used a water pre-saturation sequence with 128 averages, repetition time = 5 s and 64K time domain data points. Pre-processing of the time domain data included exponential multiplication (line broadening 0.3 Hz), Fourier transformation, zero- and first-order phase correction. TSP was used for chemical shift calibration and quantification of media and intracellular metabolites. Metabolite peak assignment was done from the literature values (Massie *et al*, 2007) and also using 2D spectra (COSY and TOCSY). Metabolite peak areas were used for estimating metabolite. Cell number was estimated from a cohort sample, and cell protein content was estimated from the cell pellet. Metabolite concentrations were normalised to the cell number.

### Glucose flux experiments using 1,2-^13^C2 glucose and GC/MS

Cells were grown in media supplemented with FBS and incubated in normoxic or hypoxic conditions for 12 h. Cells were harvested by scraping on ice, and metabolites were obtained by methanol/chloroform extraction (Wu *et al*, 2008). The aqueous phase was dried using a Speedvac and derivatised by silylation (Perroud *et al*, 2006). RNA ribose extraction and derivatisation was performed as previously described (Boren *et al*, 2003). The sample was injected into a GC-TOF MS (Leco Pegasus HT GC/TOFMS; Leco UK, Stockport, UK) and run according to methods described previously (Perroud *et al*, 2006). The obtained chromatograms were analysed using the ChromaTOF software package (Leco UK) to identify the different peaks. Mass spectral results were accepted only if the standard sample deviation was < 1% of the normalised peak intensity.

### Transfections and RNA interference

For the establishment of stable cell lines, transfections were performed using the Lonza Nucleofector system and reagents according to the manufacturer's specifications. For siRNA, cells were transfected using the Lipofectamine technology and reagents (Lipofectamine2000, Invitrogen 11668-019) or the Lonza Nucleofector. Scramble (1027280) and HIF1A (SI02664053 and SI02664431) siRNAs were obtained from Qiagen.

### Chromatin immunoprecipitation

Chromatin immunoprecipitation (ChIP) was performed as previously described (Schmidt *et al*, 2009). See Supplementary Materials and Methods for details.

### ChIP-seq Solexa library preparation

Single-end Solexa sequencing libraries were prepared as previously described. See Supplementary Materials and Methods for details.

### Microarray analysis

Expression analysis was carried out on the Illumina BeadChip platform. Quality control, pre-processing and quantile normalisation were carried out in R using the Bioconductor package beadarray (Dunning *et al*, 2007). Differential expression analysis was carried out with the Bioconductor package limma (Smyth *et al*, 2004). Genes were said to be differentially expressed at a 1% false discovery rate (Benjamini & Hochberg, 1995). See Supplementary Materials and Methods for details.
