# Supplementary material for: Nuclear ARRB1 induces pseudohypoxia and cellular metabolism reprogramming in prostate cancer
Source: EMBO J. 2014 May 16;33(12):1365–82. doi: 10.15252/embj.201386874 (PMC4194125; doi:10.15252/embj.201386874)
Supplement: Supplementary file 1 [file embj0033-1365-sd1.pdf]

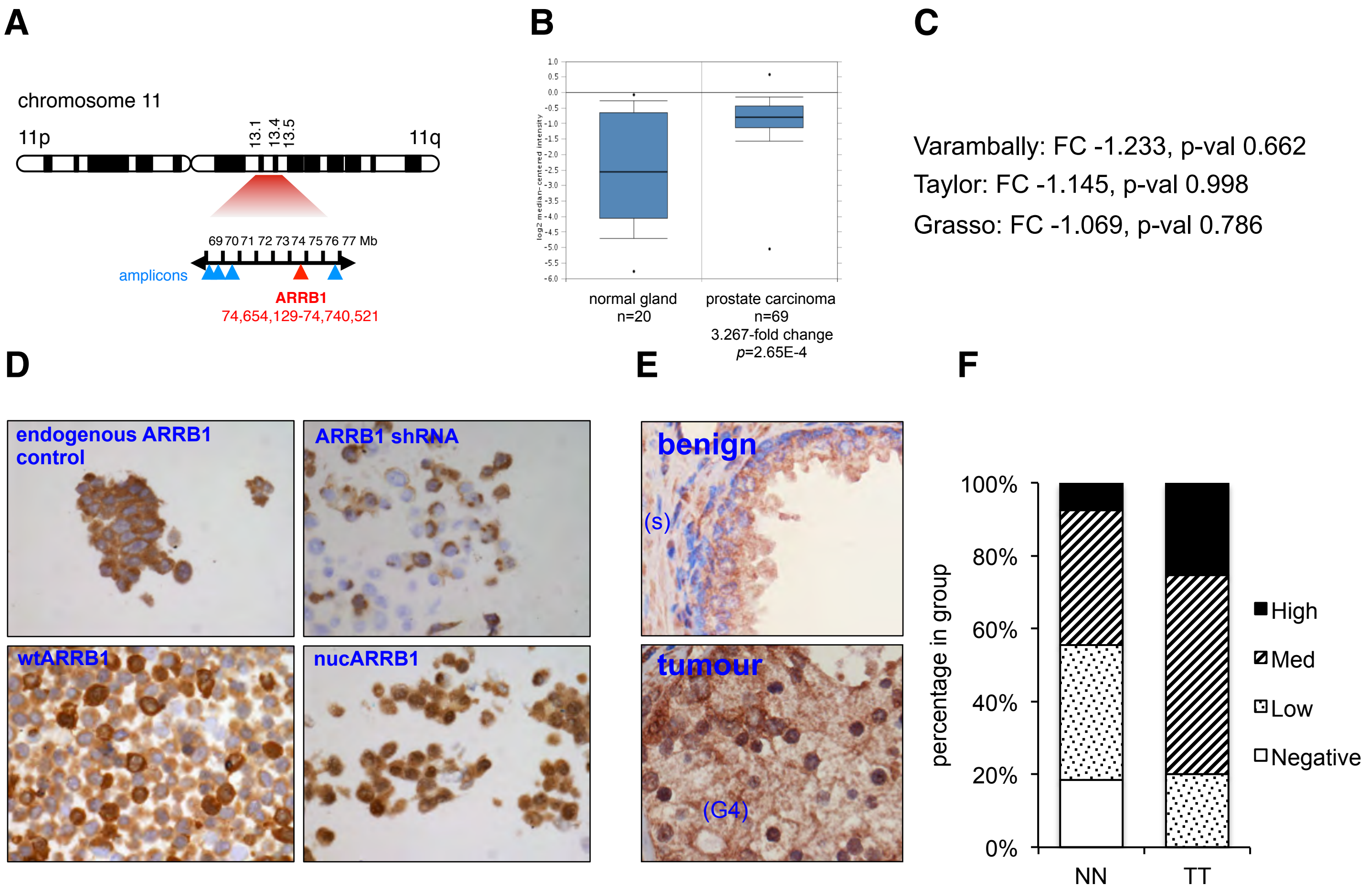

Supplemental Figure S1. ARRB1 expression levels in clinical samples of prostate cancer.

A. Mapping of ARRB1 to chromosome 11. Blue arrows indicate regions frequently amplified in cancers.

B. Using Oncomine, we probed four independent clinical studies (Wallace, Varambally, Taylor and Grasso) for differential expression of ARRB1 between normal and cancer tissue. Apart from Wallace Prostate (B), which showed a strong and very significant upregulation of ARRB1 in cancer *vs* normal tissue (3.267-fold change, *p*=2.65E-4), Varambally, Grasso and Taylor Prostate studies (C) showed a very small reduction in ARRB1 expression. However, as indicated by the high p-value, the effect observed in these three studies is not significant and could reflect a high degree of heterogeneity within a particular dataset.

D. Validation of ARRB1 immunostaining in cell lines expressing endogenous levels of ARRB1, reduced levels of ARRB1 (ARRB1 shRNA) or overexpressing wtARRB1 or nucARRB1. Wt and nucARRB1 show higher expression levels compared to control. While wtARRB1 localises mainly to the cytoplasm, nucARRB1-overexpressing cells, as expected, show strong nuclear staining. ARRB1 knockdown shows reduced staining intensity.

E. Representative expression pattern of ARRB1 in normal and malignant prostate cancer tissues. Benign tissue shows weak nuclear and moderate cytoplasmic staining in luminal and basal cells with staining also present in stromal cells (s). Moderate to intense cytoplasmic and intense nuclear staining is noted in Gleason 4 (G4) areas of the tumour.

F. Quantification of ARRB1 staining in normal and malignant prostate tissue showed in d.
