# Supplementary material for: Nuclear ARRB1 induces pseudohypoxia and cellular metabolism reprogramming in prostate cancer
Source: EMBO J. 2014 May 16;33(12):1365–82. doi: 10.15252/embj.201386874 (PMC4194125; doi:10.15252/embj.201386874)
Supplement: Supplementary file 2 [file embj0033-1365-sd2.pdf]

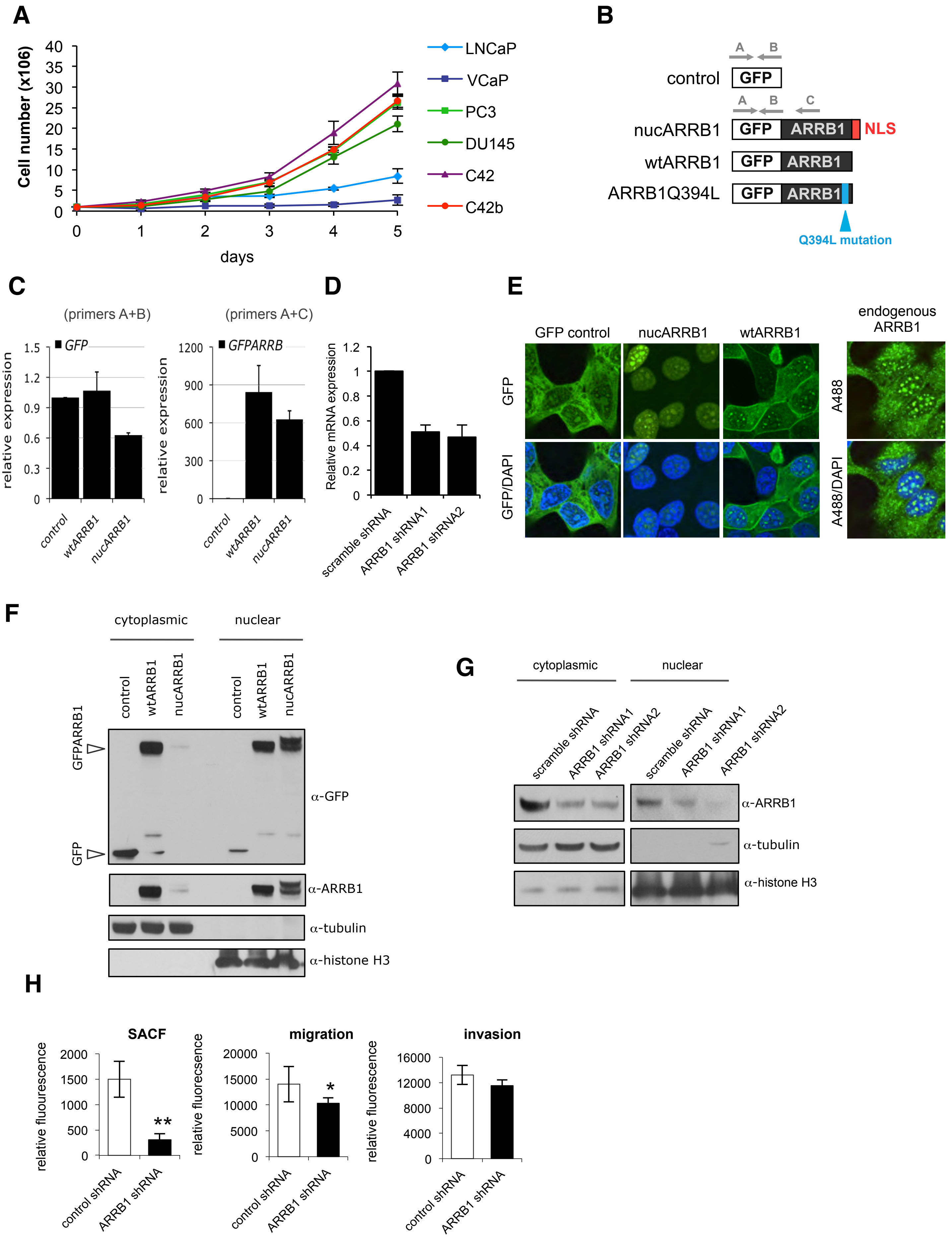
A
Cell number (x106)
days
LNCaP
VCaP
PC3
DU145
C42
C42b
B
control
GFP
nucARRB1
ARRB1
NLS
wtARRB1
ARRB1Q394L
Q394L mutation
C
(primers A+B)
(primers A+C)
GFP
GFPARRB
relative expression
control
wtARRB1
nucARRB1
D
Relative mRNA expression
scramble shRNA
ARRB1 shRNA1
ARRB1 shRNA2
E
GFP control
nucARRB1
wtARRB1
endogenous ARRB1
GFP
GFP/DAPI
A488
A488/DAPI
F
cytoplasmic
nuclear
GFPARRB1
α-GFP
α-ARRB1
α-tubulin
α-histone H3
G
cytoplasmic
nuclear
α-ARRB1
α-tubulin
α-histone H3
H
SACF
migration
invasion
relative fluourescence
relative fluorecsence
relative fluorescence
control shRNA
ARRB1 shRNA

Supplemental Figure S2. Characterization of ARRB1 overexpressing and ARRB1 knock-down (KD) cell-lines used in this study.

A. Comparison of the growth rates of a panel of prostate cancer cell lines expressing different levels of ARRB1 (see Figure 2A).

B. GFP control, nucARRB1, wtARRB1 and Q394LARRB1 constructs used in the generation of stable cell-lines in this study. The primers (A, B and C) locations used for measuring the expression levels of the various constructs are highlight on the constructs schematics. NLS=nuclear localization signal. Q394LARRB1 mutant construct was previously characterized[32,33].

C-D. Expression levels of exogenous ARRB1 constructs (wtARRB1 and nucARRB1) *vs* control (GFP) and endogenous (scramble control) *vs* ARRB1 knockdown (shRNA1 and 2) as determined by qRT-PCR using the primer pairs highlighted in B.

E. Confocal sections showing GFP fluorescence in GFP control and GFP-tagged ARRB1 expressing cells (left) and endogenous ARRB1 in parental C4-2s (right, primary=A1CT anti-ARRB, secondary=A488-conjuguated anti-rabbit). WtARRB1 localises to the membrane, cytoplasm and nucleus, whereas nucARRB1 is solely nuclear with a pattern very similar to that of the nuclear fraction of the endogenous protein. A488 and GFP=green, DAPI=blue.

F. Immunoblot of the cytoplasmic/nuclear fractions in endogenous and stable ARRB1-overexpressing cell-lines. Tubulin and Histone H3 were used as markers for cytoplasmic and nuclear fractions, respectively.

G. Immunoblot of cytoplasmic/nuclear fractions in control and two different ARRB1 shRNA cell-lines. Tubulin and Histone H3 were used as markers for cytoplasmic and nuclear fractions, respectively.

H. SACF (Soft Agar Colony Formation, i.e. anchorage-independent growth), migration and invasion of nucARRB1 *vs* control of scramble shRNA *vs* ARRB1 shRNA. n=6, values are mean ± s.e.m., *=p-value<0.05, **=p-value<0.01.
