# Supplementary material for: Nuclear ARRB1 induces pseudohypoxia and cellular metabolism reprogramming in prostate cancer
Source: EMBO J. 2014 May 16;33(12):1365–82. doi: 10.15252/embj.201386874 (PMC4194125; doi:10.15252/embj.201386874)
Supplement: Supplementary file 3 [file embj0033-1365-sd3.pdf]

A

number of binding sites

ChIP	endoARRB1	nucARRB1	tissue
ARRB1	11129	14916	7530
p300	41727	52180	-
H3K4me1	129397	117859	71104
H3K4me3	27347	25552	24180
RNAPolII	68667	43603	-

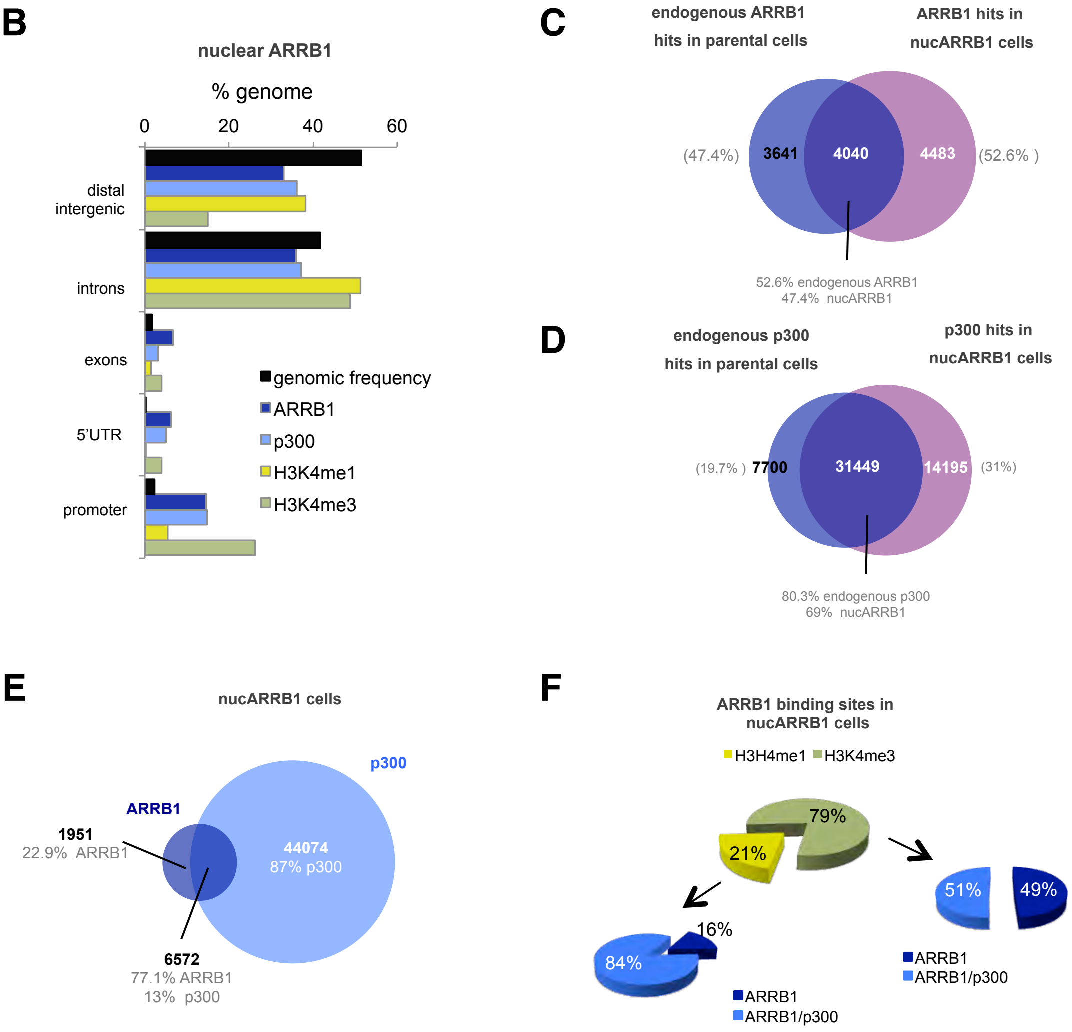

Supplemental Figure S3. Genomic landscape of cells stably overexpressing nuclear ARRB1.

A. Number of MACS peaks for ARRB, p300, H3K4me1, H3K4me3 and RNAPII ChIPs in parental C4-2, stable nucARRB1 C4-2s and human prostate tissue.

B. Genomic distribution generated using CEAS of ARRB1, p300, H3K4me1 and H3K4me3 in nucARRB1 C4-2 cells compared to the genomic frequency of the regions considered.

C. Venn diagram showing the overlap (minimum 1bp) between ARRB peaks in parental C4-2 (endogenous ARRB1) and nucARRB1 cells.

D. Venn diagram showing the overlap (minimum 1bp) between p300 peaks in parental C4-2 (endogenous ARRB1) and nucARRB1 cells.

E. Venn diagram showing the overlap (minimum 1bp) between functional endogenous ARRB1 and p300 binding sites in nucARRB1 cells.

F. Distribution of ARRB1 binding sites, either alone or shared with p300, relative to enhancer (H3K4me1) and promoter (H3K4me3) regions in nucARRB1 cells.
