# Supplementary material for: Nuclear ARRB1 induces pseudohypoxia and cellular metabolism reprogramming in prostate cancer
Source: EMBO J. 2014 May 16;33(12):1365–82. doi: 10.15252/embj.201386874 (PMC4194125; doi:10.15252/embj.201386874)
Supplement: Supplementary file 4 [file embj0033-1365-sd4.pdf]

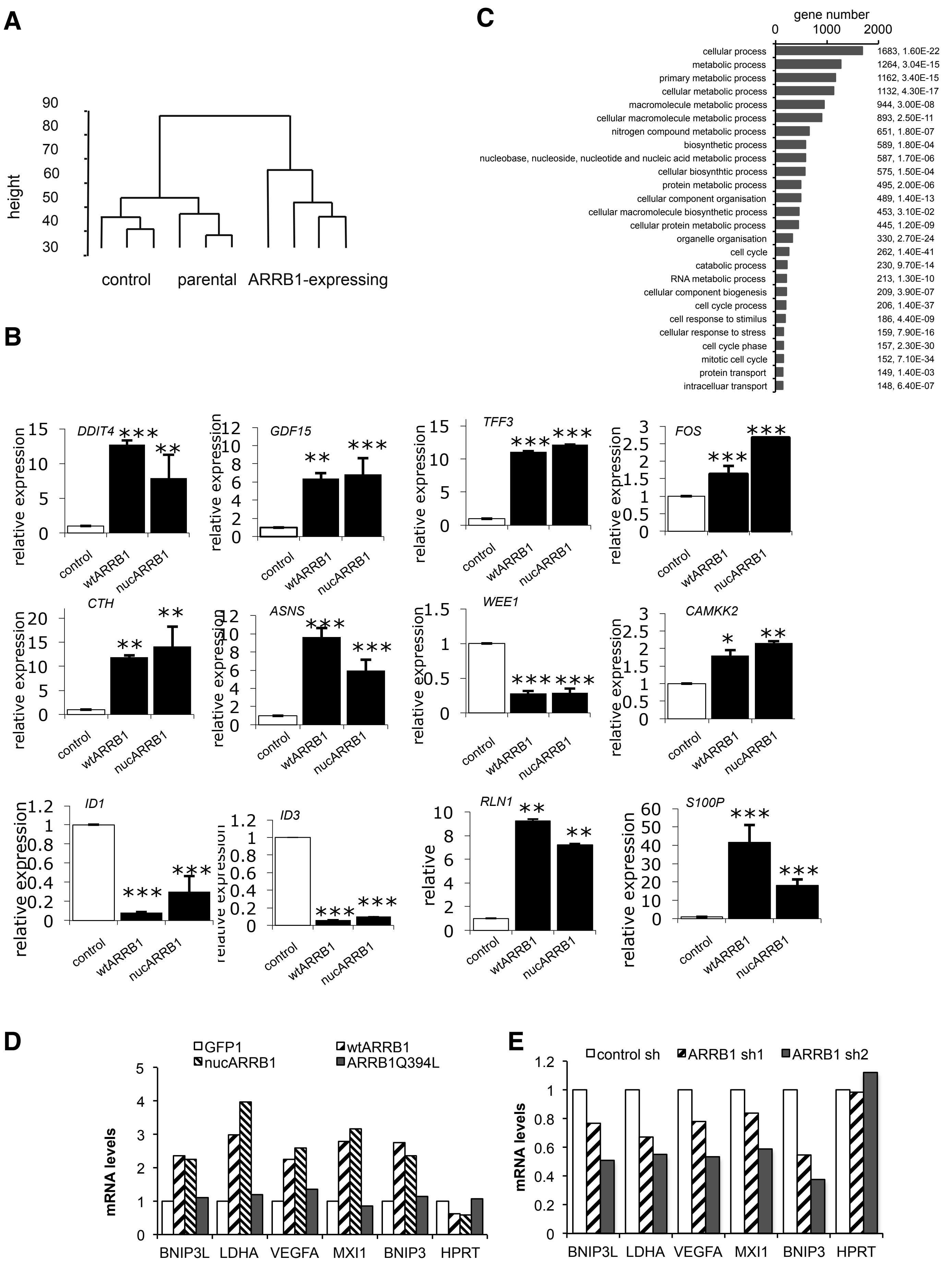
A
height
90
80
70
60
50
40
30
control
parental
ARRB1-expressing
C
gene number
0
1000
2000
cellular process 1683, 1.60E-22
metabolic process 1264, 3.04E-15
primary metabolic process 1162, 3.40E-15
cellular metabolic process 1132, 4.30E-17
macromolecule metabolic process 944, 3.00E-08
cellular macromolecule metabolic process 893, 2.50E-11
nitrogen compound metabolic process 651, 1.80E-07
biosynthetic process 589, 1.80E-04
nucleobase, nucleoside, nucleotide and nucleic acid metabolic process 587, 1.70E-06
cellular biosynthtic process 575, 1.50E-04
protein metabolic process 495, 2.00E-06
cellular component organisation 489, 1.40E-13
cellular macromolecule biosynthetic process 453, 3.10E-02
cellular protein metabolic process 445, 1.20E-09
organelle organisation 330, 2.70E-24
cell cycle 262, 1.40E-41
catabolic process 230, 9.70E-14
RNA metabolic process 213, 1.30E-10
cellular component biogenesis 209, 3.90E-07
cell cycle process 206, 1.40E-37
cell response to stimilus 186, 4.40E-09
cellular response to stress 159, 7.90E-16
cell cycle phase 157, 2.30E-30
mitotic cell cycle 152, 7.10E-34
protein transport 149, 1.40E-03
intracelluar transport 148, 6.40E-07
B
DDIT4
GDF15
TFF3
FOS
CTH
ASNS
WEE1
CAMKK2
ID1
ID3
RLN1
S100P
relative expression
control
wtARRB1
nucARRB1
D
GFP1
wtARRB1
nucARRB1
ARRB1Q394L
mRNA levels
BNIP3L
LDHA
VEGFA
MXI1
BNIP3
HPRT
E
control sh
ARRB1 sh1
ARRB1 sh2
mRNA levels
BNIP3L
LDHA
VEGFA
MXI1
BNIP3
HPRT

Direct transcriptional targets of ARRB1.

In order to narrow down our search for direct transcriptional targets of ARRB1, we integrated the ChIP data and gene expression profiling obtained in nucARRB1 cells. Rather than setting an arbitrary threshold, we used GSEA (Subramanian et al., 2005) to identify the genomic distance for which the correlation between altered gene expression (ranked by increasing p-value using $-\log_2$[p-value]) and the binding of ARRB1 is optimal. We used sliding windows of increasing size around the ARRB1 binding sites limiting ourselves to a maximum of 20kb since. Whilst more distal sites could regulate gene expression, the technical challenges of employing unbiased chromosome conformation capture to identify these sites lie beyond the scope of this study. We found that the 10kb window generated the most significant enrichment in ARRB1-regulated genes. Using this window, we derived a core set of 854 potential direct transcriptional target genes of ARRB1 (Table 1B).

F

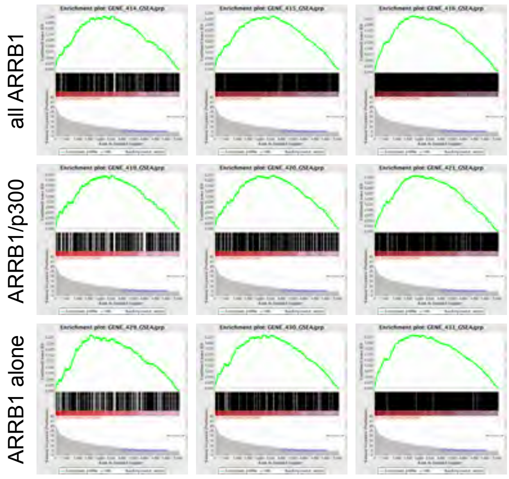

G

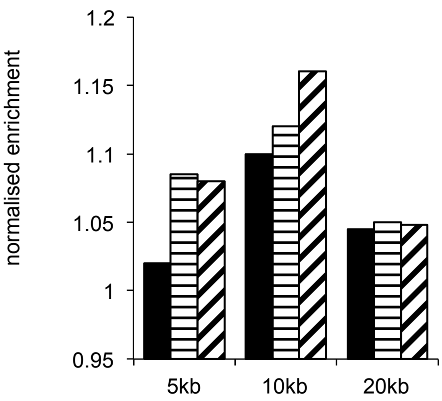

H

Category	Count	%	p-value	FDR
Translation	44	5.378973105	4.68E-10	8.22E-07
cellular process	543	66.38141809	7.21E-10	1.28E-06
cellular metabolic process	374	45.72127139	5.33E-09	9.47E-06
cell cycle	72	8.80195599	1.12E-08	1.99E-05
translational elongation	21	2.567237164	2.24E-08	3.98E-05
DNA replication	29	3.54232274	3.61E-05	6.41E-05
metabolic process	414	50.61124694	6.06E-08	1.08E-04
cell cycle process	56	6.84596577	7.30E-08	1.30E-04

I

Top Canonical Pathways

Name	p-value	Ratio
EIF2 Signaling	1.3E-06	22/202 (0.109)
Hypoxia Signaling in the Cardiovascular System	1.72E-04	10/66 (0.152)
mTOR Signaling	2.41E-04	18/211 (0.085)
Prostate Cancer Signaling	3.8E-04	11/98 (0.112)
Mitotic Roles of Polo-Like Kinase	8.02E-04	9/69 (0.13)

J

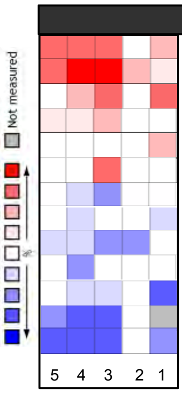

Gene	median rank	p-value	log2FC in nucARRB1
TFF3	421	1.74E-6	+2.34
GDF15	745	5.84E-6	+2.65
ATF3	1164	2.53E-4	+2.04
CTH	3717	0.015	+3.21
IDH1	8981	0.166	+1.30
ERN1	7566	0.181	+1.01
GATA2	6696	0.125	-0.80
IDH2	7192	0.118	-1.52
ID2	3482	0.003	-0.96
DNAJC9	4913	0.060	-1.53
GAPDH	4472	0.050	-0.87
ID3	1037	3.49E-6	-2.89
ID1	509	3.26E-8	-2.66

1.Yu, J Clin Oncol (2004)
2.Wallace, Cancer Res (2008)
3.Varambally, Cancer Cell (2005)
4.Taylor, Cancer Cell (2010)
5.Grasso, Nature (2012)

Supplemental Figure S4. Transcriptional landscape of ARRB1 in prostate cancer cells.
A. Gene probes from gene expression profiling in parental C4-2, GFP control, wtARRB1 and nucARRB1 cell-lines were grouped into clusters of similar expression profiles. Both parental C4-2 and GFP control cells grouped in close adjacent clusters whereas wtARRB1 and nucARRB1 (collectively named ARRB1-expressing) were mixed in a separate branch.
B. Validation of the gene expression array experiment. Sixty-two differentially regulated transcripts were chosen and qRT-PCR was preformed on total mRNA samples extracted from GFP-, wtARRB1- or nucARRB1-expressing C4-2 cells. Out of these, 61 confirmed the expression levels reported in the gene expression array. The results of 12 different transcripts are shown here.
C. Functional analysis of the nucARRB1 transcriptome using DAVID Gene Ontology analysis showing an enrichment of genes involved in cellular metabolism and cell cycle. Gene number and p-value are shown on the right of the graph.
D-E. Expression levels of targets genes common to ARRB1 and HIF1A by qPCR in GFP control *vs* wtARRB1, nucARRB1 and ARRB1Q394L or ARRB1 KD *vs* control. For all graphs, values are means ± s.e.m. of 3 replicates. *p-value<0.05, **p-value<0.01, ***p-value<0.001.
F. GSEA plots of normalized enrichment of the genes with altered expression in nucARRB1 that have an ARRB1 binding site within 5, 10 or 20kb of their TSS.
G. Histogram of the normalized enrichment maxima from the plots above plotted for each condition.
H. DAVID gene ontology analysis of the direct transcriptional targets of ARRB1.
I. IPA analysis of the ARRB1 direct transcriptional targets showing the associated top canonical pathways.
J. Summary of gene expression data from five separate studies using clinical samples showing the expression of multiple direct ARRB1 targets in prostate cancer compared with benign (represented as the percentile rank for each gene in each set and labeled with median gene rank and median *P*-value for each gene over all nine studies; data from Oncomine).
