# Supplementary material for: Nuclear ARRB1 induces pseudohypoxia and cellular metabolism reprogramming in prostate cancer
Source: EMBO J. 2014 May 16;33(12):1365–82. doi: 10.15252/embj.201386874 (PMC4194125; doi:10.15252/embj.201386874)
Supplement: Supplementary file 5 [file embj0033-1365-sd5.pdf]

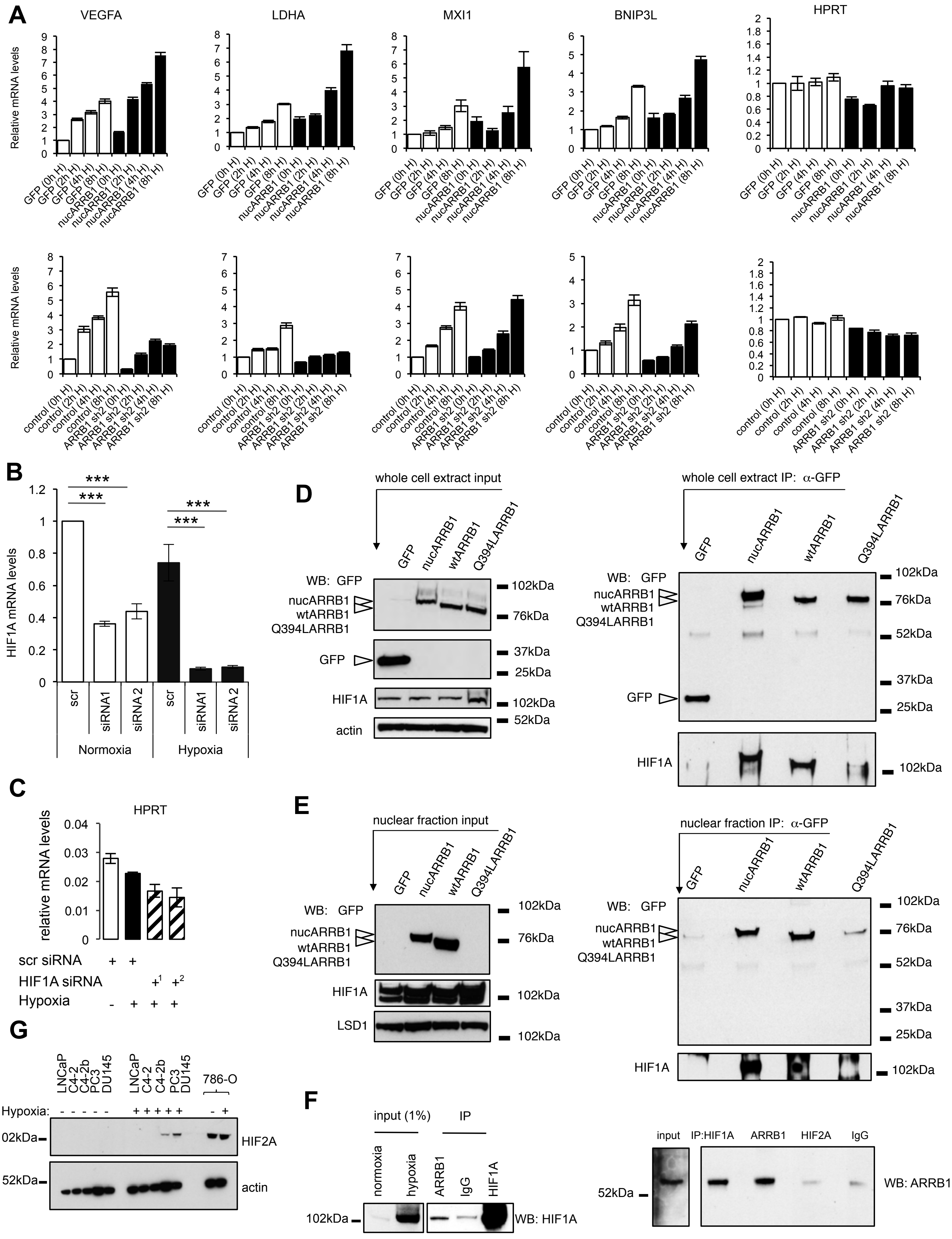

A
VEGFA
LDHA
MXI1
BNIP3L
HPRT
Relative mRNA levels
GFP (0h H)
GFP (2h H)
GFP (4h H)
GFP (8h H)
nucARRB1 (0h H)
nucARRB1 (2h H)
nucARRB1 (4h H)
nucARRB1 (8h H)
control (0h H)
control (2h H)
control (4h H)
control (8h H)
ARRB1 sh2 (0h H)
ARRB1 sh2 (2h H)
ARRB1 sh2 (4h H)
ARRB1 sh2 (8h H)
B
HIF1A mRNA levels
***
scr
siRNA1
siRNA 2
Normoxia
Hypoxia
C
HPRT
relative mRNA levels
scr siRNA
HIF1A siRNA
Hypoxia
D
whole cell extract input
whole cell extract IP: α-GFP
GFP
nucARRB1
wtARRB1
Q394LARRB1
WB: GFP
HIF1A
actin
102kDa
76kDa
52kDa
37kDa
25kDa
E
nuclear fraction input
nuclear fraction IP: α-GFP
LSD1
F
input (1%)
IP
normoxia
hypoxia
ARRB1
IgG
HIF1A
WB: HIF1A
input
IP:HIF1A
ARRB1
HIF2A
IgG
WB: ARRB1
G
LNCaP
C4-2
C4-2b
PC3
DU145
786-O
Hypoxia:
HIF2A
actin

**H**

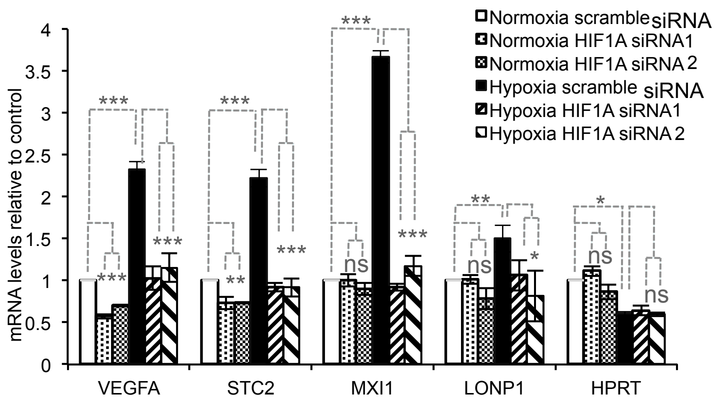


**I**

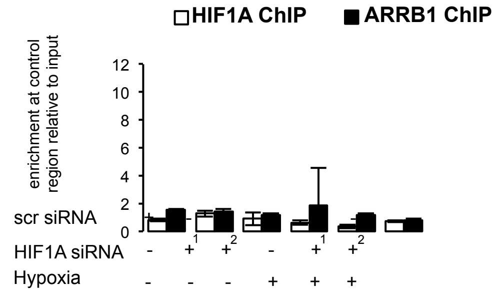


**Supplemental Figure S5. ARRB1 interacts with HIF1A to modulate its transcriptional activity.**

**A.** GFP control and nucARRB1 (top) or scramble shRNA and ARRB1 shRNA (bottom) cells were incubated in hypoxia (1% $O_2$) for 0, 2, 4 or 8 hours. Expression levels of target genes and control (HPRT) were measured by qRT-PCR. A heatmap version of this experiment is shown in Figure 5A.

**B.** Validation of HIF1A KD in C4-2 cells. Cells were transfected with two different HIF1A siRNAs, harvested 48 hours after transfection and HIF1A expression levels were determined following KD.

**C.** ARRB1 and HIF1A recruitment at the control region used in the ChIP experiment detailed in Figure 5D.

**D-E.** GFP, nucARRB1, wtARRB1 and Q394LARRB1 cells were grown in hypoxia (1% $O_2$ for 12h) to stabilize HIF1A. Whole cell extracts (wce) or nuclear fraction were prepared and used to co-immunoprecipitate ARRB1-associated complexes using an anti-GFP antibody. The immunocomplexes were analysed by immunoblotting using a different antibody against GFP or against HIF1A. Wce inputs (D, left) show expression of GFP, nucARRB1, wtARRB1 and Q394LARRB1 as well HIF1A stabilisation. Actin was used as loading control. Nuclear extracts inputs (E, left) show enrichment in constructs that are found abundantly in the nucleus (nucARRB1 and wtARRB1); whereas, as expected, Q394LARRB1 is absent from the nuclear compartment. HIF1A stabilisation is shown and LSD1 was used as loading control. Immunoblots of the pulled-down protein complexes are shown on the right. Wce co-IPs show all GFP-tagged (GFP, nucARRB1GFP, wtARRB1GFP and Q394LARRB1GFP) to be pulled-down (top blot). The bottom immunoblot shows HIF1A to be present in the nucARRB1 and wtARRB1 fractions (and to a much lower extend in the Q394LARRB1 fraction, possibly due to small leakage in the nucleus) but absent in the control GFP extract. Nuclear extracts co-IPs (E, blots on the right) show the nucleus-expressed constructs (nucARRB1 and wtARRB1) only to be pulled-down (compared to all constructs pulled-down in wce; see D right-hand-side blot). The bottom blot shows HIF1A to be co-IPed with nucARRB1 and wtARRB1 only and absent from the GFP control extract.

**F.** Parental C4-2 cells were grown in hypoxia (1% $O_2$ for 12h) to stabilize HIF1A. Left: immunoprecipitation of protein complexes from nuclear extracts was performed using anti-ARRB1, anti-HIF1A or IgG antibodies and the immunocomplexes were analysed by immunoblotting using an antibody against HIF1A. Inputs (1% of the total nuclear extracts) are shown on the left. Right: immunoprecipitation of protein complexes from nuclear extracts was performed using anti-ARRB1, anti-HIF1A, anti-HIF2A or IgG antibodies and the immunocomplexes were analysed by immunoblotting using an antibody against ARRB1. Input (1% of the total nuclear extracts) is shown on the left. The immunoblots shows ARRB1 to be present in complexes pulled-down with ARRB1 and HIF1A but not HIF2A or IgG control.

**G.** HIF2A protein levels was assessed in a panel of prostate cancer cell lines under normoxic and hypoxic (1% $O_2$ for 8hrs) conditions. 786-O *VHL*-/- RCC cells where used as positive control. Due to the *VHL* deletion, these cells show high levels of HIF2A. HIF2A levels are below the detection threshold in all prostate cancer cell lines under normoxic conditions. Only hypoxic DU145s and, to a lesser extend, PC3s show some degree of HIF2A stabilisation, albeit a lot less than that seen in 786-O control cells.

**H.** Validation of some of the HIF1A transcriptional targets in C4-2: in order to ensure our cell-line responds to hypoxic treatment, HIF1A knockdown and validate our transcriptional read-out, C4-2 cells were transfected with either scramble siRNA or two different HIF1A siRNAs. 48 hours after transfection, the cells were then incubated in normoxia or hypoxia (1% $O_2$ for 8h) to induce HIF1A transcriptional response. Expression levels of HIF1A targets were determined by qRT-PCR. HIF1A KD inhibits hypoxia-induced transcriptional activation of the targets, thus confirming them as HIF1A targets in prostate cancer C4-2 cells. *HPRT* was used as negative control.

**I.** HIF1A and ARRB1 recruitment to a control chromatin was assessed by ChIP followed by qRT-PCR on nucARRB1 cells transfected with two different HIF1A siRNAs. Fourty-eigth hours after transfections, the cells were incubated in normoxia (N) or hypoxia (H, 1% $O_2$ for 12hrs). Enrichment of ARRB1 and HIF1A normalized to input and chromatin amounts at the control site. No effect was observed at this control region.
