# Supplementary material for: Nuclear ARRB1 induces pseudohypoxia and cellular metabolism reprogramming in prostate cancer
Source: EMBO J. 2014 May 16;33(12):1365–82. doi: 10.15252/embj.201386874 (PMC4194125; doi:10.15252/embj.201386874)
Supplement: Supplementary file 6 [file embj0033-1365-sd6.pdf]

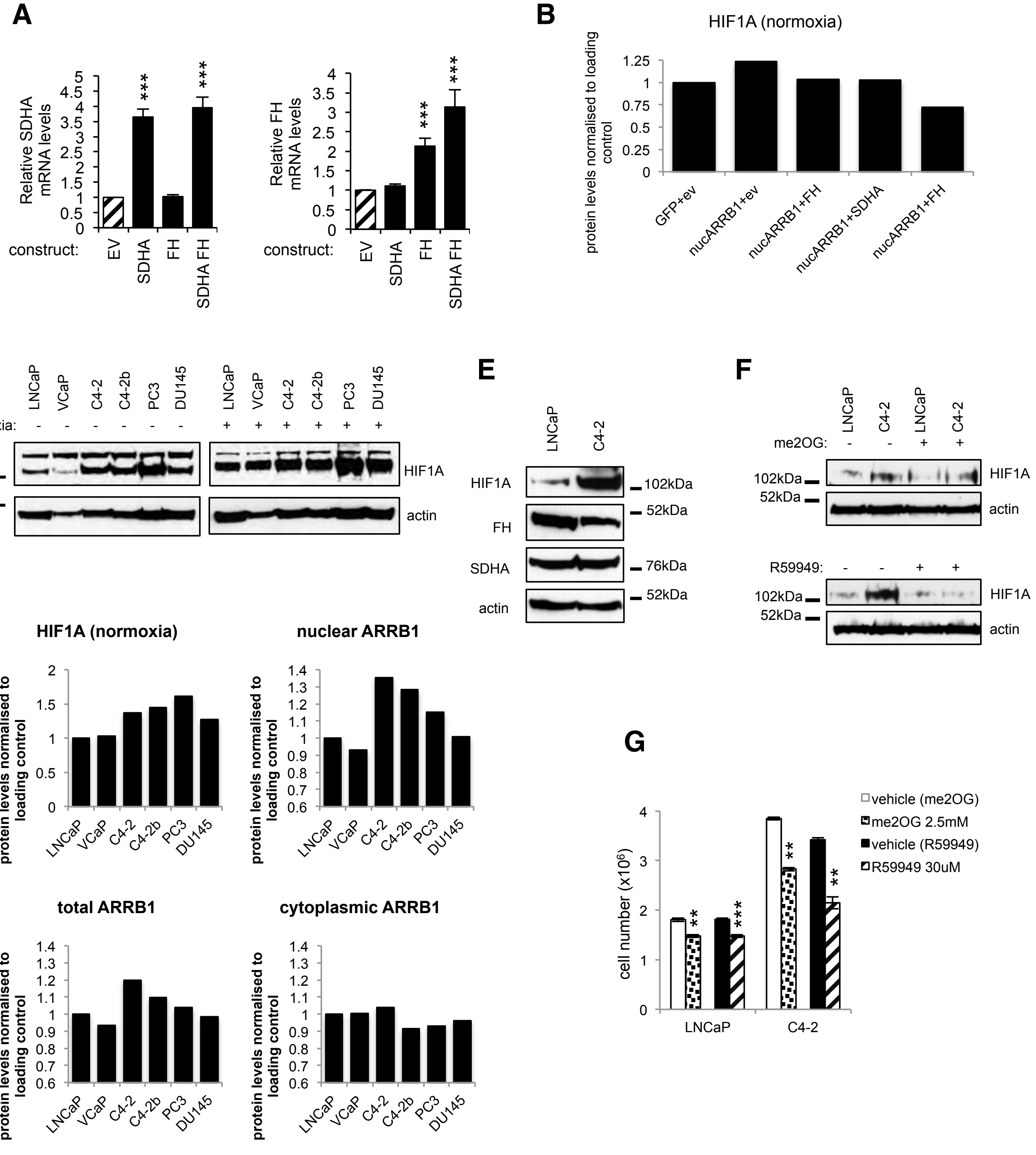
A
Relative SDHA mRNA levels
construct:
EV
SDHA
FH
SDHA FH
***
Relative FH mRNA levels
B
HIF1A (normoxia)
protein levels normalised to loading control
GFP+ev
nucARRB1+ev
nucARRB1+FH
nucARRB1+SDHA
C
LNCaP
VCaP
C4-2
C4-2b
PC3
DU145
Hypoxia:
102kDa
52kDa
HIF1A
actin
D
nuclear ARRB1
total ARRB1
cytoplasmic ARRB1
E
FH
SDHA
76kDa
F
me2OG:
R59949:
G
cell number (x10^6)
vehicle (me2OG)
me2OG 2.5mM
vehicle (R59949)
R59949 30uM
**
***

Supplemental Figure S6. Nuclear ARRB1 levels correlate with cell sentivity to pharmacological HIF1A inhibition

A. Relative *SDHA* (left) and *FH* (right) mRNA levels in nucARRB1 transiently transfected with empty vector control (ev), SDHA, FH or equal amounts of SDHA and FH expression vectors. GFP control cells transfected with ev were used a control and baseline for HIF1A expression levels.

B. Quantification of the HIF1A protein levels in the blot showed in A. Expression of FH or SDHA in nucARRB1 cells lowers HIF1A expression levels to levels similar to that seen in GFP with ev control. Combined expression of FH and SDHA in nucARRB1 result in a 40% reduction in the levels of HIF1A (compared to nucARRB1+ev) and a 30% reduction comapred to GFP+ev control.

C. HIF1A protein levels was assessed by immunoblotting in a panel of prostate cancer cell lines under normoxic and hypoxic (1% O_2 for 8hrs) conditions.

D. Quantification of the HIF1A and ARRB1 (total, nuclear or cytoplasmic) protein levels in a panel of prostate cancer cells. The blot in C was used to quantify HIF1A levels and the blot on Figure 2A was used to quatify the levels of ARRB1 in the same cell lines. Nuclear ARRB1 levels correlate with the levels of HIF1A i.e. lines with low nuclear ARRB1 also display the lowest levels of HIF1A. Cytoplasmic levels of ARRB1 do not show any correlation with HIF1A levels.

E. HIF1A, FH and SDHA protein expression levels were determined by immnublotting in low (LNCaP) and high (C4-2) ARRB1-expressing cell extracts.

F. LNCaPs and C4-2 were treated with PHD activators me2OG (2.5mM) and R59949 (30uM) and HIF1A protein levels were determined by immunoblot after 2 days.

G. LNCaP and C4-2 cell numbers 3 days post-treatment with me2OG or R59949.
