# Supplementary material for: Nuclear ARRB1 induces pseudohypoxia and cellular metabolism reprogramming in prostate cancer
Source: EMBO J. 2014 May 16;33(12):1365–82. doi: 10.15252/embj.201386874 (PMC4194125; doi:10.15252/embj.201386874)
Supplement: Supplementary file 7 [file embj0033-1365-sd7.pdf]

A

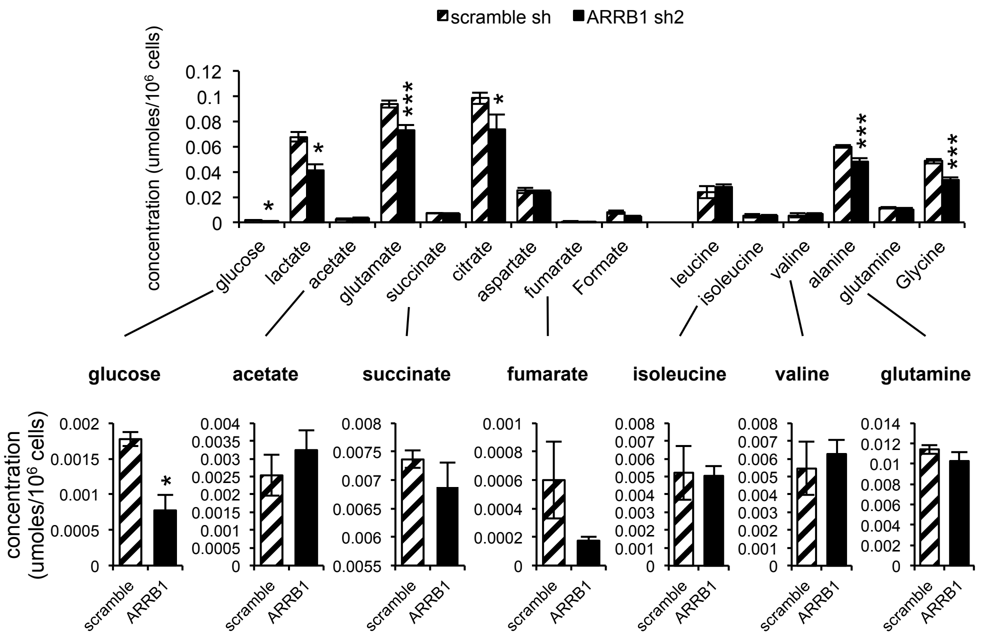

B

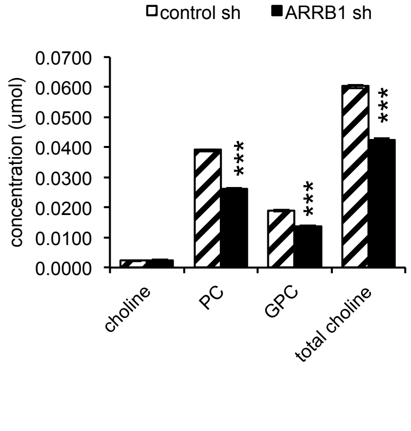

Supplemental Figure S7. ARRB1 regulates metabolism in prostate cancer cells.

A. ^{1}H NMR Proton nuclear magnetic resonance measurements of intracellular glucose and lactate, amino acids and TCA intermediate in shARRB1 cells compared to scramble control. **B.** Kennedy pathway phospholipid intermediate metabolites choline, phosophocholine (PC), glycerophosphocholine (GPC) and total choline in shARRB1 cells lysates compared to scramble control. N=3, values are mean ± s.e.m.
