# Supplementary material for: Nuclear ARRB1 induces pseudohypoxia and cellular metabolism reprogramming in prostate cancer
Source: EMBO J. 2014 May 16;33(12):1365–82. doi: 10.15252/embj.201386874 (PMC4194125; doi:10.15252/embj.201386874)
Supplement: Supplementary file 9 [file embj0033-1365-sd9.pdf]

SUPPLEMENTAL EXPERIMENTAL METHODS

Sequence read analysis. Sequence reads were generated by the Illumina analysis pipeline version 1.3.4 and 1.4.0. The two lanes of reads were combined for each sample, and aligned to the Human Reference Genome (assembly hg18, NCBI Build 36.1, March 2008) using MAQ (Li et al., 2008). Next they were filtered by alignment quality score, removing all reads with a MAQ score less than 20, and exact duplicate reads were removed such that no single read start position was represented more than once. Enriched regions of the genome were identified by comparing the ChIPed samples to Input samples using the MACS peak calling algorithms (Zhang et al., 2008). All ChIP-seq data have been deposited at the NCBI Short Read Archive and identified consensus binding sites (peaks) are available in Supplementary data. Overlap, subtraction, union and feature annotation of ChIP-seq enriched regions were done using the Galaxy website (Taylor et al., 2007). Genomic distribution of ARRB ChIP peak sequences was done using CEAS.

Illumina beadarrays. Total RNA samples were harvested from parental or stable C4-2 cells grown in RPMI supplemented with 10% FBS. These comprised: three GFP control lines; three parental C4-2 lines; two wtARRB1 lines and two nucARRB1 lines. Total RNA was extracted using Qiagen RNeasy Plus according to the manufacturer's instructions. Quality control was performed with an Agilent Bioanalyser. cDNA was generated and biotin labelled using the Illumina TotalPrep RNA Amplification Kit, according to the manufacturers instructions. Hybridization and scanning were performed using Standard Illumina protocols.

Autocorrelation analysis of Illumina gene expression data. The Illumina HumanWG v2 BeadArrays consist of two replicate sections that we treat as technical replicate arrays for the purposes of this analysis due to small but systematic shifts between sections that need to be addressed in the normalization. Data were analysed from the raw bead-level using the beadarray software, with spatial artefacts identified and removed automatically (BASH) and curated manually. The resultant, reduced, data set was then summarized in a standard fashion (with outliers removed) in order to obtain a mean log-intensity and standard error for each probe/array combination. The November 2008 annotation from http://www.compbio.group.cam.ac.uk/Resources/Annotation/index.html was used to map probes to transcripts, and probes with no "bad" match were discarded along with those that registered no signal above background on all arrays. To detect probes that showed a systematic, smooth, change over time without prescribing a form for that change we used the autocorrelation at lag 1 as a measure of activity. This measure identifies profiles where neighbouring time-points are more similar than disparate time points, and so can identify all smooth and systematic gene expression changes regardless of the shapes of their profiles. To account for the uncertainty in our measurements, we simulated 100 sets of observations from the known means and standard errors, calculated the autocorrelation of each and took the mean. Simulations, and arguments of symmetry suggested that a cut-off of autocorrelation=0.5 would lead to a low false-discovery rate. Standard clustering methods were then used to group probes that passed the false-discovery rate threshold into clusters of similar expression profiles (Supplemental Fig S4A).

Co-immunoiprecipitation. For endogenous ARRB1-HIF1A co-immunoprecipitation, C4-2 cells grown in normoxic or hypoxic (1% O_2 for 12h) conditions were washed once with ice-cold PBS, and pelleted by centrifugation at 4000 rpm for 10 minutes at 4°C. Nuclear fractions were isolated as described previously (Schmidt et al., 2009) with the following modifications. Briefly, cell pellets were resuspended in lysis buffer 1 (50 mM Hepes-KOH pH 7.5, 140 mM NaCl, 1 mM EDTA, 10% glycerol, 0.5% IGEPAL® CA-630 (Sigma-Aldrich), 0.25% Triton X-100 and protease inhibitor cocktail (Roche)), incubated with rotation for 10 minutes at 4°C and centrifuged at 2500 rpm for 3 minutes at 4°C, followed by incubation for 5 minutes at 4°C in lysis buffer 2 (10 mM Tris pH 7.5, 200 mM NaCl, 1 mM EDTA, 0.5 mM EGTA and protease inhibitor cocktail). Nuclei were pelleted by centrifugation at 2500 rpm for 3 minutes at 4°C, incubated with rotational mixing for 30 minutes at 4°C in lysis buffer 3 (50 mM Tris-HCL pH 7.5, 5%

glycerol, 150 mM NaCl, 5 mM EDTA pH 8.0, 15 mM $MgCl_2$, 1% IGEPAL® CA-630, 0.10% sodium deoxycholate, 20 mM N-ethylmaleimide, protease and phosphatase inhibitor cocktails (Roche)) and briefly sonicated in a probe sonicator. Co-immunoprecipitations were carried out from nuclear lysates by incubation with magnetic dynabeads (Invitrogen) covalently crosslinked to ChIP-grade antibodies against HIF1A (Novus Biologicals, rabbit polyclonal, NB100-134SS), HIF2A (AbCam ab199), ARRB1 (rabbit polyclonal, A1CT, a gift from R. Lefkowitz) or non-specific rabbit IgG (Dako) overnight at 4°C. Beads were washed three times with wash buffer (50 mM Tris-HCL pH 7.5, 5% glycerol, 150 mM NaCl, 5 mM EDTA pH 8.0, 15 mM $MgCl_2$, 0.1% IGEPAL® CA-630, 0.10% sodium deoxycholate, 20 mM N-ethylmaleimide and protease inhibitor cocktail) and proteins were eluted by incubation in *NuPAGE® LDS Sample Buffer* (Invitrogen) for 10 minutes at 70°C, followed by analysis by SDS-PAGE and Western Blotting.

For co-IP from stables cell lines, GFP control, nucARRB1, wtARRB1 and Q394LARRB1 cells grown in hypoxic (1% O_2 for 12h) conditions were washed once with ice-cold PBS, and pelleted by centrifugation at 4000 rpm for 10 minutes at 4°C. Nuclear fractions were isolated as described previously(Schmidt et al, 2009), with the same modifications as above. Co-immunoprecipitations were carried out from nuclear lysates by incubation with GFP antibody (Abcam ab290) covalently coupled to magnetic dynabeads (Invitrogen) overnight at 4°C. Protein complexes were washed five times in wash buffer (50 mM Tris-HCL pH 7.5, 5% glycerol, 150 mM NaCl, 5 mM EDTA pH 8.0, 15 mM $MgCl_2$, 1.5% IGEPAL® CA-630, 0.10% sodium deoxycholate, 20 mM N-ethylmaleimide supplemented with protease and phosphatase inhibitor cocktail) and bound proteins were eluted by incubation in *NuPAGE® LDS Sample Buffer* (Invitrogen) for 10 minutes at 70°C, followed by analysis by SDS-PAGE and Western Blotting using anti-GFP (AbCam, ab6556) or anti-HIF1A (Novus Biologicals, NB100-134SS).

Quantitative real-time PCR. We used quantitative real-time PCR to confirm ARRB binding sites and gene expression changes using the primers listed below, using SYBRgreen chemistry (Applied Biosystems, 2x SYBRgreen master mix) in an ABI7900 instrument (Applied Biosystems).

Primers used for expression qRT-PCR: ARRB1-F (TTTGTGGCCAACGTACAGTG), ARRB1-R (GTGAAAGGGTAAGCGTGCTC), ASNS-F (GGAGCCTTTTCTTCCTGGAC), ASNS-R (GGGTACATCCCGACAGTGAT), CAMKK2-F (TGAAGACCAGGCCCGTTTCTACTT), CAMKK2-R (TGGAAGGTTTGATGTCACGGTGGA), CTH-F (CCGTTCTGGAAATCCCACTA), CTH-R (GTGGCTGCTAAACCTGAAGC), DDIT4-F (GACTGAACTTTTGGGGTGGA), DDIT4-R (TAGGTGGCTGCCTCAGTTTT), FOS-F (CTCCAGTGCCAACTTCATTC), FOS-R (CAGCCATCTTATTCCTTTCC), GDF15-F (ACAATCCCATGGTGCTCATT), GDF15-R (TATGCAGTGGCAGTCTTTGG), GFP-F (CAACTACAACGCCCACAATG), GFP-R (GTTGTGGCGGATCTTGAAGT), GFPARRB1-F (CACGGCATGGATGAGCTG), GFPARRB1-R (AAACACTCTTGGTGCCTTTGTC), HPRT-F (TTGCTTTCCTTGGTCAGGCA), HPRT-R (GCTTGCGACCTTGACCATCTT), ID1-F (GACTTTAGGGGGTGGGATTC, ID1-R (CTGAGAAGCACCAAACGTGA), ID3-F (CTGGACGACATGAACCACTG), ID3-R (GTAGTCGATGACGCGCTGTA), RPLP0-F (ATCAACGGGTACAAACGAGTC), RPLP0-R (CAGATGGATCAGCCAAGAAGG), S100P-F (GATGCCGTGGATAAATTGCT), S100P-R (ACTTGTGACAGGCAGACGTG), STC2-F (TTCCCCGGATTTAGCTTTTT), STC2-R (CCGGGTTCCCTTTTACTCTC), TFF3-F (GTCTGGGAGCTTGACAAAGG), TFF3-R (TGCTCTGGATTGTTTGCTTG), WEE1-F (ACCTCGGATACCACAAGTGC), WEE1-R (AGTGCCATTGCTGAAGGTCT), RLN1-F (CCAAATGGAAGGACGATGTT), RNL1-R (GAGACCTTTTGCTCCAGGTG), LONP1-F (ATCAGCAGACAGCTGGAGGT), LONP1-R (GTCCTCCGCCTCCTTCTT), STC2-F (TACCTCAAGCACGACCTGTG), STC2-R (GGAGCAAGTCCTTGAAATGG)MXI1-F (CTCCCATGAAGCCTTTTGAA), MXI1-R (AGCTGGGTGCCTAGTGCTAA), VEGFA-F (TCTTCAAGCCATCCTGTGTG), VEGFA-R (TGGTGATGTTGGACTCCTCA), HIF1A-F (CCACCTATGACCTGCTTGGT), HIF1A-R (TATCCAGGCTGTGTCGACTG), BNIP3L-R (CTACCCATGAACAGCAGCAA), BNIP3L-R (CATGTCTCCATTGTGGATGG), BNIP3-F (CCCATAGCATTGGAGAGAAAA), BNIP3-R (CCGACTTGACCAATCCCATA), SDHA-F (ATTTGGTGGACAGAGCCTCA), SDHA-R (AGGTGTGCAATAGCGAGTGG).

Primers used for *VEGFA* , *MXI1* and control promoter ChIP RT-PCR:

MXI1-L-F (AGAAGAAAGGTCCCGATTGC), MXI1-L-R (CCTGAAGACCTGGGGTATCA), control-F (CATCTCCTCCCACTTCTGGA), control-R (TGTGTGCCTTCACACAGTCA), VEGFA (CTAGCACCAGCGCTCTGTC), VEGFA-R (CCCGATCAATGAATATCAAA)
