# Supplementary material for: Nuclear ARRB1 induces pseudohypoxia and cellular metabolism reprogramming in prostate cancer
Source: EMBO J. 2014 May 16;33(12):1365–82. doi: 10.15252/embj.201386874 (PMC4194125; doi:10.15252/embj.201386874)
Supplement: Supplementary file 10 [file embj0033-1365-sd10.pdf]

Transparent process (uncropped, unalterd scanned blots)
Supplemental Figure S2

Figure S2F

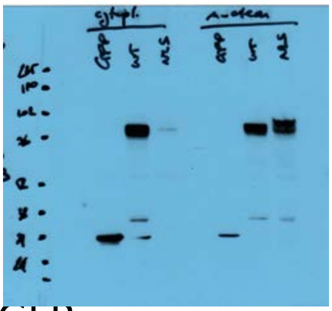

GFP

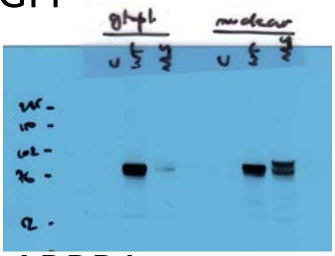

ARRB1

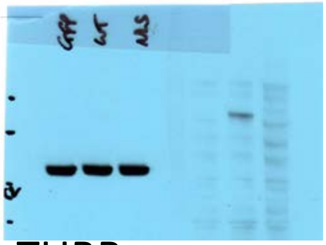

TUBB

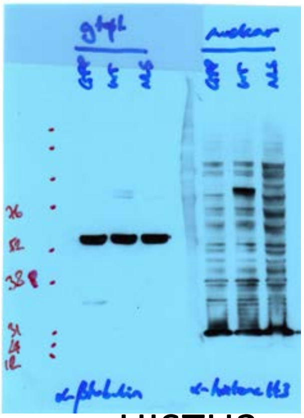

HISTH3

Figure S2G

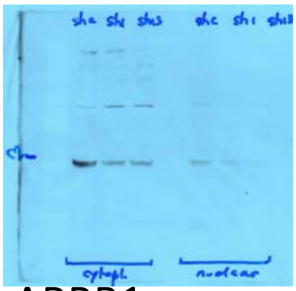

ARRB1

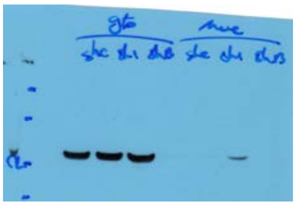

TUBB

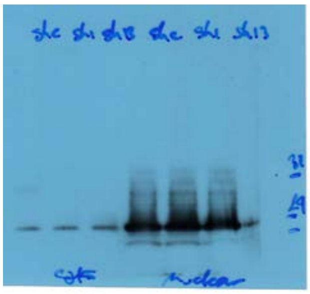

HISTH3
