# Supplementary material for: Nuclear ARRB1 induces pseudohypoxia and cellular metabolism reprogramming in prostate cancer
Source: EMBO J. 2014 May 16;33(12):1365–82. doi: 10.15252/embj.201386874 (PMC4194125; doi:10.15252/embj.201386874)
Supplement: Supplementary file 11 [file embj0033-1365-sd11.pdf]

Transparent process (uncropped, unalterd scanned blots)

Figure S5

Figure S5D

WCE inputs

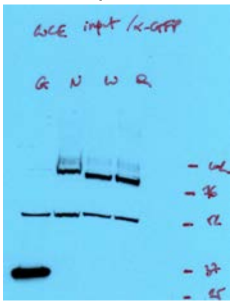

GFP

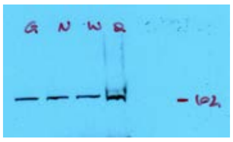

HIF1A

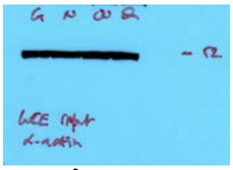

actin

WCE coIPs

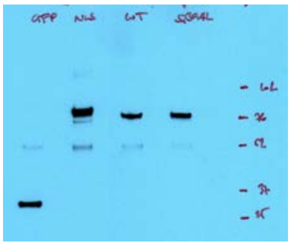

GFP

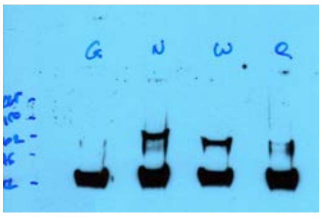

HIF1A

Figure S5E

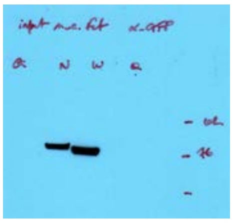

GFP

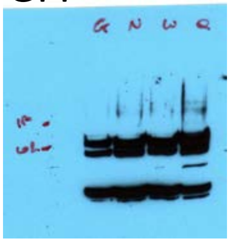

HIF1A

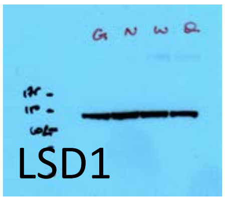

LSD1

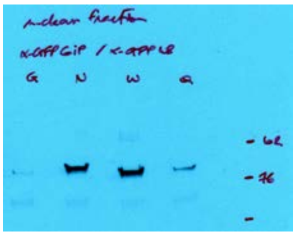

GFP

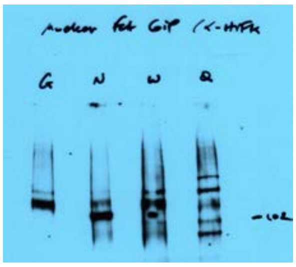

HIF1A

Figure S5F

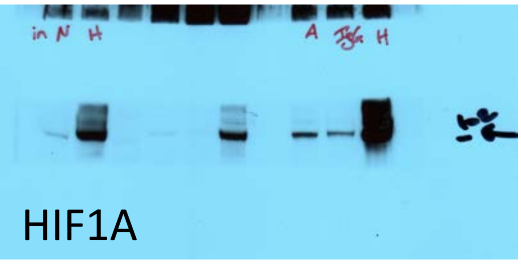

HIF1A

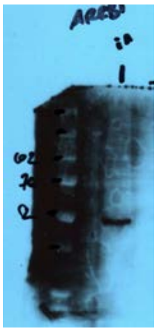

ARRB1

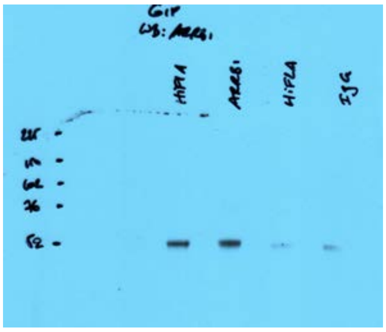

ARRB1

Figure S5G

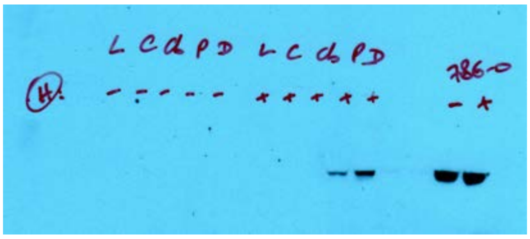

HIF2A

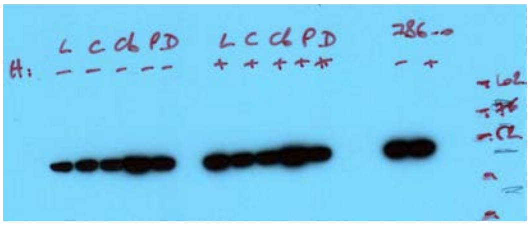

actin
