# Supplementary material for: Nuclear ARRB1 induces pseudohypoxia and cellular metabolism reprogramming in prostate cancer
Source: EMBO J. 2014 May 16;33(12):1365–82. doi: 10.15252/embj.201386874 (PMC4194125; doi:10.15252/embj.201386874)
Supplement: Supplementary file 13 [file embj0033-1365-sd13.pdf]

THE EMBO JOURNAL

Manuscript EMBO-2013-86874

Nuclear ARRB1 induces pseudohypoxia and cellular metabolism reprogramming in prostate cancer

Vincent Zecchini, Basetti Madhu, Roslin Russell, Nelma Pértega-Gomes, Anne Warren, Edoardo Gaude, Joana Borlido, Rory Stark, Heather Ireland-Zecchini, Roheet Rao, Helen Scott, Joan Boren, Charlie Massie, Mohammad Asim, Kevin Brindle, John Griffiths, Christian Frezza, David E Neal and Ian G Mills

Corresponding author: Ian Mills, University of Oslo

Review timeline:

Submission date:	11 September 2013
Editorial Decision:	01 October 2013
Revision received:	28 February 2014
Editorial Decision:	19 March 2014
Revision received:	13 April 2014
Accepted:	17 April 2014

Transaction Report:

(Note: With the exception of the correction of typographical or spelling errors that could be a source of ambiguity, letters and reports are not edited. The original formatting of letters and referee reports may not be reflected in this compilation.)

Editor: Thomas Schwarz-Romond

1st Editorial Decision 01 October 2013

I am pleased to enclose comments from two expert referees on your study. Not unexpected, the scientist mostly assessing the genome-wide occupancy data/prostate cancer angle, is rather supportive of the data, despite indicating that stronger functional, in-vivo support may further raise the studies significance.

Crucially, the referee commenting on the HIF-1/cancer metabolism aspect similarly demands much stronger support for the functional interplay between HIF-1 (possibly HIF-2) with nucARRB1 in what is proposed as pseudohypoxic HIF-regulation.

Though certainly not insisting on a complete set of in-vivo validations, definitive clarification on the major points 3, 4, 6; establishing causality as per point 7; and focusing further-reaching experiments on the points 9-11 of ref#2 should improve the significance and thus strengthen the general appeal of your dataset.

Conditioned on such amendments, I would be delighted to invite a single round of major revisions for our more general title. Please note that a subsequent assessment would possibly involve the

© EMBO

critical referee before reaching a final decision.

Please do not hesitate to get in touch regarding potential timeline/feasibility of the requested experimental expansions (preferably via E-mail).

REFEREE REPORTS:

Referee #1:

The findings from this manuscript are interesting and relevant. The quality of the data is very good and the conclusions are valid. The ChIP-seq data and the combination with gene expression changes is an important dataset and it provides biological support for the role of ARRB1 in prostate cancer. The data included in the manuscript is extremely extensive, yet all important and valuable. It would of course be interestingly to investigate the role of ARRB1 and HIF1alpha in prostate cellular migration in an in vivo model. However, considering the already extensive data in the paper, thereviewer is hesitant to suggest this.

Referee #2:

Zecchini et al. present an interesting manuscript demonstrating that increased nuclear ARRB1 in prostate cancer cells mediates a shift from oxidative to glycolytic metabolism via pseudohypoxic regulation of HIF1 . As previously described in breast cancer cells, nuclear ARRB1 interacts with HIF1 to up-regulate its transcriptional activity. This study provides evidence that nucARRB1 increases stabilisation of HIF1 via regulation of FH and SDHA. It is proposed that a combination of these effects act to promote increased glycolytic metabolism, a classical feature of cancer cells. This study is of potential interest to cancer researchers as well as biologists in the HIF/hypoxia fields. However, there are several concerns with the current study.

Major Comments

1. Control prostate tissue is referred to as 'normal' in the text, but it actually refers to the 'benign' tissue (Fig. 1b). Benign tissue is not necessarily the same as normal prostate tissue. The authors must describe what they precisely mean by 'benign'; were these 'benign' samples collected from prostate cancer patients or from individuals with normal, healthy prostate? ARRB1 expression in normal, healthy prostate tissue should be determined.
2. C4-2 cells are shown to express strong nucARRB1 level (Fig. 1g). This is, however, not consistent with the nuclear ARRB1 level shown in Suppl Fig. S1(l), which appears to indicate a markedly lower nuclear ARRB1 level relative to the cytoplasmic ARRB1.
3. The rationale for using C4-2 cells for the generation of stable lines overexpressing GFP-tagged wtARRB1 or nucARRB1 is unexplained. These stable lines are used to measure migration, invasion and colony formation to infer an association between ARRB1, in particular nucARRB1, expression and aggressiveness. However, these C4-2 cells are 'faster growing, more aggressive and highly tumorigenic and metastatic' that 'display higher nuclear levels of ARRB1 compared to the other lines.' The authors should instead perform similar experiments on low nucARRB1 expressing lines.
4. Considering the authors' contention that ARRB1 regulates HIF1 stability and activity, it will be critically important to determine HIF1 (and HIF2) protein levels in C4-2 and other prostate cancer cell lines examined in Fig. 1g.
5. What is Fig. 5a actually showing? The legend for Fig. 5a does not correspond to the figure itself or the text discussing this figure. Where is the blot of OH-HIF1A? Regardless, the authors need to first provide the expression level of nucARRB1 and provide the actual experiment using DMOG. Why was DMOG used?
6. Fig. 5d is missing the blots showing the levels of FH and SDHA pre and post knockdown. The results of Fig. 5e are rather modest; it may be statistically significant, but it remains to be determined whether such a modest effects on the indicated genes are biologically significant.
7. The authors suggest that me2-OG treatment reduces the number of C4-2 cells but not LNCaP

cells due to the influence of me2-OG to reactivate PHD and thereby modulate HIF1 stability, which influences cell growth (Fig. 5f; however in the text it is erroneously referred to Fig. 6f). The authors need to show that me2-OG reactivates PHD in their system and that it decreases HIF1 levels and that the reduction in HIF1 leads to the attenuation of cell growth. Second, perhaps more perplexing is why do you not see similar effects in LNCaP. Again, what is the level of HIF1 in these cells versus C4-2 cells (see also comment no. 4)?
8. What is the mechanism by which ARRB1 inhibits FH and SDH?
9. Fig. 4 labelling is incorrect; text, figure legends and figures themselves are inconsistent with each other. The authors knockdown HIF1A and show that it reduces the mRNA levels of VEGFA, MXI1, etc. (Fig. 4b, which should be Fig. 4c?). This raises a general question as to why HIF2 is not compensating for the reduced level of HIF1 . Does ARRB1 exclusively interact with HIF1 ?
10. The evidence support the notion that ARRB1 and HIF1 physically interact is unconvincing. The authors conclusion regarding physical interaction is based on one supplementary result (Suppl Fig. S5d). It is rather concerning that a massive pool of HIF1 co-precipitates a whisper of ARRB1. What percentage of ARRB1 is actually co-precipitating with HIF1 ? Considering the quality and the significance of this experiment, it will require critical controls beyond what is provided.
11. The authors suggest that nucARRB1 increased protein stabilisation of HIF1 as well as increasing gene transcription of HIF1 target genes via interaction between HIF1 and ARRB1 at the gene promoter. The authors should clarify whether the increased HIF1 stabilization due to increased nucARRB1 is sufficient for the changes in hypoxia-responsive gene expression without the requirement of ARRB1 binding to HIF1 .

Minor Comments

1. The title of the paper is misspelled and there are other spelling mistakes in the manuscript. For example on p. 5, 'A high proportion of ARRB1 sites (66.5%) were associated with the functional markers H3K4me1 or H3K4me1'. See also p. 13, 16 and 30.

1st Revision - authors' response 28 February 2014

Referee #1:

The findings from this manuscript are interesting and relevant. The quality of the data is very good and the conclusions are valid. The ChIP-seq data and the combination with gene expression changes is an important dataset and it provides biological support for the role of ARRB1 in prostate cancer. The data included in the manuscript is extremely extensive, yet all important and valuable. It would of course be interestingly to investigate the role of ARRB1 and HIF1alpha in prostate cellular migration in an in vivo model. However, considering the already extensive data in the paper, the reviewer is hesitant to suggest this.

We would like to thank the reviewer for the very positive response to the primary submission and hope that he/she finds the revised version even better.

Referee #2:

Zecchini et al. present an interesting manuscript demonstrating that increased nuclear ARRB1 in prostate cancer cells mediates a shift from oxidative to glycolytic metabolism via pseudohypoxic regulation of HIF1α. As previously described in breast cancer cells, nuclear ARRB1 interacts with HIF1α to up-regulate its transcriptional activity. This study provides evidence that nucARRB1 increases stabilisation of HIF1α via regulation of FH and SDHA. It is proposed that a combination of these effects act to promoteincreased glycolytic metabolism, a classical feature of cancer cells. This study is of potential interest to cancer researchers as well as biologists in the HIF/hypoxia fields. However, there are several concerns with the current study.

We would like to thank the reviewer for investing their time in reading our manuscript and for their helpful comments and suggestions. We believe that the implementation of the reviewer's comments

© EMBO

has improved this study and hope the reviewer will find our revision of the manuscript satisfactory.

Major Comments

1.Control prostate tissue is referred to as 'normal' in the text, but it actually refers to the 'benign' tissue (Fig. 1b). Benign tissue is not necessarily the same as normal prostate tissue. The authors must describe what they precisely mean by 'benign'; were these 'benign' samples collected from prostate cancer patients or from individuals with normal, healthy prostate? ARRB1 expression in normal, healthy prostate tissue should be determined.

The reviewer's comment is pertinent. Normal, healthy prostate tissue is not widely available as radical prostatectomies are performed on patients with diagnosed prostate cancer and generally not on healthy men. We do agree that the word "non-neoplastic tissue" would be more appropriate than "normal" tissue and the authors have amended the manuscript accordingly. Considering that our TMAs were constructed by our pathologists with samples from patients who underwent radical prostatectomy as a treatment for prostate cancer, the term "non-neoplastic tissue" describes the apparently healthy/benign glands that were selected adjacent to the tumours (note that cases of tumours with an infiltrative character were not used). Using the non-neoplastic tissue adjacent to malignant glands we were able, together with our pathologists, to find differences regarding ARRB1 expression, when compared to malignant glands thus demonstrating that the malignant vs non-malignant phenotype shows changes of ARRB1 expression/localization.

2. C4-2 cells are shown to express strong nucARRB1 level (Fig. 1g). This is, however, not consistent with the nuclearARRB1 level shown in Suppl Fig. S1(l), which appears to indicate a markedly lower nuclear ARRB1 level relative to the cytoplasmic ARRB1.

We understand the reviewer's comment. Figure 1 (now Figure 2A) shows the steady-state levels of ARRB1; whereas Figure S1 (now Supplemental Fig S2G) is assessing the impact of ARRB1 knock-down on ARRB1's levels and thus, the two figures are not directly comparable.

Indeed, the sub-cellular fractions from the two independent experiments in Figure 2A (endogenous cyto/nuc levels in C4-2) and Supplemental Fig S2G (cyto/nuc levels in ARRB1 knockdown in C4-2) were run on separate gels and the intensity of the bands will depend on the exposure time, which was different for the two experiments and fractions. In addition, we also noticed a slight contamination of the cytoplasmic fraction by the nuclear one (as indicated by some histone H3 signal in the cytoplasmic fraction) in Supplemental Fig S2G that might have boosted the intensity control shRNA ARRB1 cytoplasmic signal relative to the nuclear one.

From these blots, we concluded that (i) in endogenous conditions, C4-2 cells show higher nuclear ARRB1 than the other lines (and in particular LNCaPs) and (ii) ARRB1 knockdown in C4-2 is efficient as the protein levels are reduced in both sub-cellular compartments compared to control shRNA.

We hope the reviewer will be satisfied with our interpretation.

3. The rationale for using C4-2 cells for the generation of stable lines overexpressing GFP-tagged wtARRB1 or nucARRB1 is unexplained. These stable lines are used to measure migration, invasion and colony formation to infer an association between ARRB1, in particular nucARRB1, expression and aggressiveness. However, these C4-2 cells are 'faster growing, more aggressive and highly tumorigenic and metastatic' that 'display higher nuclear levels of ARRB1 compared to the other lines.' The authors should instead perform similar experiments on low nucARRB1 expressing lines.

We thank the reviewer for pointing out the need to clarify our choice of cell-line. In the main text,

© EMBO

we have now indicated the rationale behind this: "The C4-2 line was selected as its higher levels of nuclear ARRB1 were better suited for ChIP." (lines 117-118). Indeed, the low ARRB1 levels seen in LNCaP represent a major technical hurdle to ChIPping. In order to be consistent, since we sought to integrate all our genomic data, we chose to perform our gene expression profiling on the same cell line.

However, the reviewer's suggestion to perform the migration/invasion assay on a less aggressive cell line with lower levels of endogenous ARRB1 such as LNCaP was excellent. In addition to clarifying in the manuscript that expression of nuclear ARRB1 in C4-2 cells enhanced the already transformed phenotype of these cells (line 106-109: "...nucARRB1 expression in C4-2 cells enhanced the transformed phenotype of the cells as indicated by an increase in anchorage-independent growth as well as migratory and invasive potential..."), we carried out the suggested experiment and reported the results in the main text (lines: 109-112) and Figure 2E. Due to the lower basal levels of ARRB1, these cells show a stronger effect than C4-2s.

We thank the reviewer for this suggestion that, we believe, has strengthened our previous results. We have moved this important piece of data, together with the proliferation data, from the Supplementary figure to the main text in Figure 2 which now provides a general overview of the effects of increasing the levels of nuclear ARRB1 in prostate cancer cells.

4. Considering the authors' contention that ARRB1 regulates HIF1α stability and activity, it will be critically important to determine HIF1α (and HIF2α) protein levels in C4-2 and other prostate cancer cell lines examined in Fig. 1g.

The authors agree with the reviewer that, in the light of the results presented in Figure 5 onwards, assessing the correlation between ARRB1 and HIF1A is a crucial point to address. We have done so by immunoblotting cell extracts from our panel of prostate cancer cell-lines. The results are reported in the main text (lines: 294-297) and in Supplemental Figure S6B. We found HIF1A levels to correlate with nuclear ARRB1 levels in our prostate cancer cell-lines. A recent study by Ranasinghe et al. in PLoS ONE (January 16, 2013) reports a similar observation to ours for LNCaP, PC3 and DU145 cells under normoxic conditions (other prostate cancer cell lines were not assessed in that study).

With regards to HIF2A, our immunoblot shows the signal to be below detection levels in all cell-lines under normoxic condition and only present in hypoxia in PC3 s and DU145 but not in LNCaP and its derivative cell lines (C4-2s and C4-2b) used in this study. We used different anti-HIF2A antibodies (one representative blot is shown in Supplemental Fig S5G of the manuscript but would be happy to include the other blot if deemed necessary) and obtained similar results. In addition, extracts from HIF2A-expressing 786-O kidney cells were used as positive control (Supplemental Fig S5G). 786-O cells are VHL-/- and show elevated levels of HIF2A.

The role of HIF2A in cancer is well documented (such as its involvement in kidney cancer). However, to this date, little is known about the potential role HIF2A might play in prostate cancer. Although this would require a thorough investigation that is beyond the scope of this study, our results suggest that HIF2A would only play a minor role in prostate cancer and that the effect we report here is entirely mediated by HIF1A.

5. What is Fig. 5a actually showing? The legend for Fig. 5a does not correspond to the figure itself or the text discussing this figure. Where is the blot of OH-HIF1A? Regardless, the authors need to first provide the expression level of nucARRB1 and provide the actual experiment using DMOG. Why was DMOG used?

We apologize to the reviewer and the editor, as it appears some mislabeling had occurred. The reviewer is correct in pointing out the mistakes in the manuscript formatting. We have now deleted the unnecessary text in the figure legend.

We initially attempted to measure the levels of hydroxylated HIF1A using a commercially available

antibody (Cell Signaling anti-OH HIF1A 3434). HIF1A stabilization is regulated by PHDs via HIF1A hydroxylation tagging it for degradation by the proteasome. In our experiment, MG132 was used to stabilize HIF1A by inhibiting proteasome activity and thus preventing HIF1A degradation but not its hydroxylation. This resulted, as expected, in an increase in the levels of HIF1A compared to normoxic untreated conditions. DMOG (Dimethyloxalylglycine, N-(Methoxyoxoacetyl)-glycine methyl ester) is a PHD inhibitor that results in stabilization of HIF1A because of the inability to tag it with OH. Thus, lower levels of OH-HIF1A together with an increase in HIF1A protein would be expected in this situation. Optimization of the OH-HIF1A staining proved difficult and, as is clear on the immunoblot below, we were not satisfied with the results. We were unable to improve the staining to provide a result of quality that was good enough to be incorporated in our manuscript and thus decided to remove it from the initial manuscript. Unfortunately, the figure legend was not amended properly and this lead to the initial confusion. We hope the reviewer will be satisfied with our explanation and the reason behind our reluctance to include the data in the manuscript.

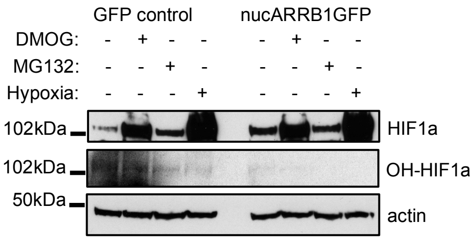

6. Fig. 5d is missing the blots showing the levels of FH and SDHA pre and post knockdown. The results of Fig. 5e are rather modest; it may be statistically significant, but it remains to be determined whether such a modest effects on the indicated genes are biologically significant.

We thank the reviewer for pointing this out. In the aforementioned experiment, FH and SDHA were overexpressed rather than knocked down and we initially provided a figure (Supplementary Figure S6A) showing the mRNA expression levels of FH and SDHA after overexpression. However, the reviewer is correct in requesting the protein levels to be assessed as well. We have therefore now provided, in addition to the mRNA expression levels (Supplemental Fig S6A), immunoblots showing the protein expression levels of FH and SDHA before and after overexpression (Figure 6I); as well as FH and SDHA expression levels in nucARRB1 vs GFP control and LNCaP vs C4-2 (Figure 6H and Supplemental Fig S6C). With regards to the targets expression, re-expression of FH and SDHA shows up to 40% reduction in mRNA for HIF1A targets (Figure 6K); a reduction that, although arguably modest, we believe reflects a true effect on transcription. To provide biological relevance to these observations, we repeated the FH/SDHA experiment, examining the effects on cell proliferation at 24, 48 and 72hrs. This showed a significant phenotype (Figure 6J) associated with the rescue that is consistent with that obtained when HIF1A is knocked down in nucARRB1 cells (Figure 6E).

7. The authors suggest that me2-OG treatment reduces the number of C4-2 cells but not LNCaP cells due to the influence of me2-OG to reactivate PHD and thereby modulate HIF1α stability, which influences cell growth (Fig. 5f; however in the text it is erroneously referred to Fig. 6f). The authors need to show that me2-OG reactivates PHD in their system and that it decreases HIF1α levels and that the reduction in HIF1α leads to the attenuation of cell growth. Second, perhaps more perplexing is why do you not see similar effects in LNCaP. Again, what is the level of HIF1α in these cells versus C4-2 cells (see also comment no. 4)?

We apologise for the legend mislabeling. Regarding this experiment, the authors would like to point

out the lower levels of ARRB1 in LNCaP vs C4-2 but should have made clearer that, as a consequence of our hypothesis, HIF1A activity would be enhanced in C4-2s vs LNCaPs and thus, C4-2s should be more sensitive than LNCaPs to HIF1A inhibition (upon reactivation of PHDs). We have made this point clearer (lines 294-301) in the text. We also provide immunoblots showing the levels of HIF1A in LNCaP and C4-2 (Supplemental Fig S6B-C). In addition, following the reviewer's advice, we also assessed HIF1A protein levels upon treatment with the drugs and provide immunoblots showing HIF1A destabilisation upon me2OG treatment (Supplemental Fig S6D). In addition, we treated the cells with R59949, another HIF1A inhibitor. This resulted in a similar effect to me2OG both on HIF1A stability and on cell proliferation (Supplemental Fig S6D-E).

8. What is the mechanism by which ARRB1 inhibits FH and SDH?

The authors believe this occurs as an indirect effect of nuclear ARRB1 on both FH and SDHA transcription (resulting in downregulation of both FH and SDHA expression) (Discussion section, lines 377-380).

9. Fig. 4 labelling is incorrect; text, figure legends and figures themselves are inconsistent with each other. The authors knockdown HIF1A and show that it reduces the mRNA levels of VEGFA, MXI1, etc. (Fig. 4b, which should be Fig.4c?). This raises a general question as to why HIF2α is not compensating for the reduced level of HIF1α. Does ARRB1 exclusively interact with HIF1α?

The authors apologize for the mislabeling of figures/figure legends and have amended these accordingly as pointed out by the reviewer.

We agree with the reviewer that the role of HIF2A in our system and a potential interaction between ARRB1 and HIF2A should have been investigated. We have now addressed this point by 1) determining the expression levels of HIF2A and 2) determining if the two proteins co-immunoprecipitate in our system.

HIF1A and HIF2A are closely related and both activate hypoxia-mediated HRE-dependent transcription (Wenger et al., FASEB J., 2002). However, they have been shown to play non-redundant roles and their inactivation results in different phenotypes (Rosenberg et al., J. Am. Soc. Nephro., 2002; Wenger et al., FASEB J., 2002; Holmquist-Mengelbier et al., Cancer Cell, 2006). Several studies suggest that HIF1A and HIF2A have different transcriptional targets. In addition, HIF1A and HIF2A appear to have distinct roles in promoting the growth of different tumours. Particularly relevant to this study is the fact that the expression of genes involved in the glycolytic pathway appears to be regulated by HIF1A but not by HIF2A (Hu et al., Cell. Biol., 2003). This, together with our findings that HIF2A is expressed at very low levels in our system (HIF2A expression levels were below detection levels in our panel of prostate cancer cells, except for hypoxic PC3 and DU145; see point 4 above and Supplemental Figure S5G), supports the observation that, in our study, HIF2A may not be able to compensate for the reduced levels of HIF1A following knockdown.

Since HIF2A expression levels appear to be low, unsurprisingly we also found no physical interaction between ARRB1 and HIF2A using co-IP (see point 10 below and Supplemental Figure S5F).

These issues have been clarified in the main text (lines 236-245).

10. The evidence support the notion that ARRB1 and HIF1α physically interact is unconvincing. The authors conclusion regarding physical interaction is based on one supplementary result (Suppl

Fig. S5d). It is rather concerning that a massive pool of HIF1α co-precipitates a whisper of ARRB1. What percentage of ARRB1 is actually co-precipitating with HIF1α? Considering the quality and the significance of this experiment, it will require critical controls beyond what is provided.

Our hypothesis for an interaction between ARRB1 and HIF1A is based on previously published evidence in breast cancer cells (Shenoy et al., Oncogene, 2011). Because such an interaction, albeit in a different system, was already reported, the authors opted to include this data, although important for the present study, in the supplemental material. The co-immunoprecipitations performed in our study on endogenous and overexpressed nuclear ARRB1 also appear to confirm an interaction in prostate cancer cells. We took on board the reviewer's suggestion to try to improve the efficiency of our co-immunoprecipitation and have repeated the experiment providing a comprehensive analysis of the physical interaction between ARRB1 and HIF1A. We used whole cell extracts as well as nuclear extracts from GFP control, nucARRB1, wtARRB1 and Q394LARRB1 (a protein that is located solely in the cytoplasm as a result of a previously characterized mutation that excludes it from the nucleus; see refs 31 and 32 in the main text; see Scott et al., 2002 and Wang et al., 2003) to pull down interactions between ARRB1 and HIF1A in various cellular compartments using an anti-GFP antibody. Our experiment shows the constructs to be expressed in, as well as pulled down from, the expected cellular compartments. We show that HIF1A interacts with constructs that are expressed in both nuclear and cytoplasmic (nucARRB1 and wtARRB1) but not with the cytoplasmic only construct (Q394LARRB1, see blot Supplemental Fig S6D-E) indicating that the interaction is restricted to the nucleus. GFP was used as a control and shows no interaction.

Co-immunoprecipitation in endogenous conditions was repeated after optimization and confirms the physical interaction between ARRB1 and HIF1A under these conditions. We have also assessed the interaction between ARRB1 and HIF2A as requested by the reviewer (see point 9 above) and found there to be none.

11. The authors suggest that nucARRB1 increased protein stabilisation of HIF1α as well as increasing gene transcription of HIF1α target genes via interaction between HIF1α and ARRB1 at the gene promoter. The authors should clarify whether the increased HIF1α stabilization due to increased nucARRB1 is sufficient for the changes in hypoxia-responsive gene expression without the requirement of ARRB1 binding to HIF1α.

The point raised by the reviewer is interesting and the reviewer's request for a clarification of this point is appropriate. In order to answer it, it would be necessary to disconnect the two activities of nuclear ARRB1 i.e. 1) its effect on HIF1A protein stability (which we believe to be the result of indirect regulation of FH and SDHA expression, ultimately leading to PHD inhibition and HIF1A stabilization); and 2) its gene transcription regulation activity. However, since the nuclear localization of ARRB1 is required for HIF1A stabilization, it is difficult to conclude whether HIF1A could still activate hypoxia-mediated transcription of its targets without ARRB1. The ARRB1 KD experiment (Figure 5A) suggests that in the absence of ARRB1 (or low levels), transcription levels of HIF1A targets are lower compared to the GFP control, suggesting that, although it might not completely abolish the transcriptional hypoxic response, low ARRB1 levels do prevent their full activation. However, when ARRB1 is overexpressed, we hypothesize that it acts as a scaffold that bridges distal and proximal regulatory elements, thus enhancing HIF1A signaling. An in-depth study of such a potential transcriptional function is clearly beyond the scope of this study and we are currently preparing another manuscript documenting this aspect of ARRB1 activity using the genomic data collected during this study.

Since ARRB1's two activities (HIF1A stabilization and gene expression regulation) both appear to depend on gene transcription regulation, a meticulous dissection the various domains of ARRB1 and/or HIF1A would be required to show 1) whether these two activities might be encoded by different regions of ARRB1 and 2) whether mutations in these domains would affect the activities separately. As these potential regions are not known, we believe this extensive work, although very

informative, lies beyond the scope of this study that focuses on the metabolic downstream effect of increased nuclear ARRB1 in the context of prostate cancer.

Minor Comments

1.The title of the paper is misspelled and there are other spelling mistakes in the manuscript. For example on p. 5, 'A highproportion of ARRB1 sites (66.5%) were associated with the functional markers H3K4me1 or H3K4me1'. See also p. 13, 16 and 30.

The manuscript has been checked for spelling and labeling.

2nd Editorial Decision 19 March 2014

Your revised study has been re-assessed by one of the original referees. While generally appreciative of the provided amendments, there remain a few critical items that I would like you to address before being able to offer formal publication.

-Most importantly, a side-by side comparison of HIF1alpha- and ARRB1 expression level (most ideally quantified westerns) should be presented as to enable assessment of their relative level in various cell lines both under normoxic and hypoxic conditions (additional comment 1).

-While nuclear ARBB1 appears to rather indirectly affect FH/SDHA expression, please make sure that the blots illustrating this regulation (Fig 6H versus control lanes 1 and 2 in 6I) are consistent in their documentation.

-Please offer some clarification on point 3 (individual versus additive effect of FH/SDHA on HIF1-alpha level versus target gene regulation).

Finally, please be prepared to provide SOURCE DATA, particularly for electrophoretic gels/blots/micrographs, as The EMBO Journal aims to make primary data more accessible and transparent to the reader. This entails presentation of un-cropped/unprocessed scans for published work. We would be grateful for one PDF-file per figure with such information. These will be linked online as supplementary "Source Data" files.

On this note, we realized that the background pattern of the empty panels in figure 2A (cytoplasmic histone H3 versus nuclear b-tubulin blots) appear remarkably similar. While purely cosmetic, please double-check the provided panels.

I look forward to what should amount to the last necessary, though highly relevant amendments to your study. Please submit a finalised version addressing these crucial points using the link enclosed below to your earliest convenience.

REFEREE REPORT:

Referee #2:

Zecchini et al have sufficiently addressed many of the comments. However, there remains some concerns and ambiguities in this manuscript. This reviewer remains concerned about the lack of experimental rigor that addresses the notion that nucARRB1 affects HIF1a stability by indirectly promoting the suppression of FH and SDHA transcription.

1. One of the main points that this manuscript expresses is that nucARRB1 regulates both HIF1 stability and transcriptional activity. However, there remains no analysis in this manuscript that demonstrates HIF1 expression alongside nucARRB1 expression. Although it is mentioned in the text, figure S6B highlights only HIF1 expression in the different cell lines in normoxia and hypoxia,

but does not give the reader any appreciation of the relative expression of ARRB1 in these cell lines. Presumably ARRB1 fluctuates in a similar manner as HIF1 in these normoxic prostate cancer cells. This figure would offer further clarity to the reader as to how the expression of nucARRB1 and HIF1 are related as well as provide supportive evidence of the mechanism.

2. It remains unclear how ARRB1 inhibits FH and SDH. The authors postulate that nucARRB1 indirectly affects both FH and SDHA transcription. This is a speculation that should have been experimentally addressed.

3. The authors overexpress FH, SDHA and FH+SDHA. Figure 6I shows HIF1 expression is only affected when both FH and SDHA are overexpressed in nucARRB1+ cells. Despite this, suppression of HIF1 responsive genes reach significance in cells with either FH or SDAH over expressed.

2nd Revision - authors' response 13 April 2014

Referee #2:

Zecchini et al have sufficiently addressed many of the comments. However, there remains some concerns and ambiguities in this manuscript. This reviewer remains concerned about the lack of experimental rigor that addresses the notion that nucARRB1 affects HIF1a stability by indirectly promoting the suppression of FH and SDHA transcription.

We would like to thank the referee for the positive overall response to our revisions. Please find our responses to the remaining comments below.

1.One of the main points that this manuscript expresses is that nucARRB1 regulates both HIF1α stability and transcriptional activity. However, there remains no analysis in this manuscript that demonstrates HIF1α expression alongside nucARRB1 expression. Although it is mentioned in the text, figure S6B highlights only HIF1α expression in the different cell lines in normoxia and hypoxia, but does not give the reader any appreciation of the relative expression of ARRB1 in these cell lines. Presumably ARRB1 fluctuates in a similar manner as HIF1α in these normoxic prostate cancer cells. This figure would offer further clarity to the reader as to how the expression of nucARRB1 and HIF1α are related as well as provide supportive evidence of the mechanism.

We agree with the reviewer that correlation of the levels of ARRB1 and HIF1A in the panel of prostate cancer cell lines used in this study would offer further clarity and support our mechanism.To address this, we have determined the levels of HIF1A (under both normoxic and hypoxic conditions) in the same panel of prostate cancer cell lines that we used to determine the ARRB1 levels (see Figure 2A, cytoplasmic/nuclear fractionation, immunoblot anti-ARRB1) and quantified the expression levels of HIF1A and ARRB1. This data is now presented in Supplemental Figure S6C (immunoblot from extracts of the same cell lines that were used to measure ARRB1 levels in Figure 2A) and in Supplemental Figure S6D (histograms showing the expression levels of HIF1A and ARRB1). As predicted and in corroboration with the other results in our study, these data show that HIF1A levels correlate with nuclear ARRB1 levels (but not cytoplasmic ARRB1).

2. It remains unclear how ARRB1 inhibits FH and SDH. The authors postulate that nucARRB1 indirectly affects both FH and SDHA transcription. This is a speculation that should have been experimentally addressed.

ARRB1 has no predicted direct DNA binding domains and like many such transcriptional coregulators may therefore mediates its effects through association with a range of other proteins. Since no binding sites for ARRB1 were detected in the proximal regulatory regions of both FH and

© EMBO

SDHA but that nuclear ARRB1 levels affect the expression levels of these two genes, we believe this occurs as an indirect effect likely involving yet unidentified different transcription factors. The systematic, unbiased dissection of the precise composition of these complexes at specific binding sites impacting on the transcription of specific genes, remains largely beyond the capability of the field and therefore beyond the scope of this study. A method was recently published called RIME (rapid immunoprecipitation mass spectrometry of endogenous proteins – Mohammed et al., 2013 Cell Reports). This however does not allow for the site specific characterisation of protein complexes but rather proteomics on the ChIPped sample. Biotinylated oligonucleotides designed using site sequence information can be used as baits bit are devoid of native chromatin context. Whilst these challenges persist it is therefore apparent to us that a meaningful attempt to characterise ARRB1-associated complexes will require multiple approaches and needs to form a distinct future study owing to its difficult and challenging nature. We have documented the effect of ARRB1 on FH and SDHA in the figure legends as well as in the main text.

3. The authors overexpress FH, SDHA and FH+SDHA. Figure 6I shows HIF1α expression is only affected when both FH and SDHA are overexpressed in nucARRB1+ cells. Despite this, suppression of HIF1α responsive genes reach significance in cells with either FH or SDAH over expressed.

In order to clarify that particular point we provide a quantification of the HIF1A protein levels shown in Figure 6I (see Supplemental Figure S6B). This indicates that the higher HIF1A levels observed in nuclear ARRB1 (nucARRB1+ev well compared to control GFP+ev) are lowered to levels similar to that seen in control (GFP+ev well) upon re- expression of FH or SDHA and further reduced upon combined expression of both FH and SDHA. This effect mirrors that seen with expression levels of HIF1A target genes following re-expression of FH/SDHA (Figure 6K). We anticipate that, in nucARRB1 cells, re-expression of FH or SDHA would not completely lift, but more likely alleviate, the bottleneck effect that results in altered metabolites levels. Indeed, in the situation when only one enzyme (either FH or SDHA) is re-expressed, the levels of the other one still remain low and thus, the flux through this part of the TCA cycle is still slowed down, albeit to a lesser degree than in nucARRB1. This results in a rescue of the observed phenotype. However, when both enzymes are re-expressed, the flow through the TCA cycle is restored. Since the expression levels of exogenous FH and SDHA (see Figure 6I, second and third panels) are much higher than the endogenous levels (GFP+ev lane), the intermediate metabolites succinate and fumarate are metabolized at a higher rate, resulting in the additive effect observed.

© EMBO
