# Supplementary material for: Nuclear ARRB1 induces pseudohypoxia and cellular metabolism reprogramming in prostate cancer
Source: EMBO J. 2014 May 16;33(12):1365–82. doi: 10.15252/embj.201386874 (PMC4194125; doi:10.15252/embj.201386874)
Supplement: Supplementary file 14 [file embj0033-1365-sd14.pdf]

Transparent process (uncropped, unalterd scanned blots)
Figure 2

## Figure 2A

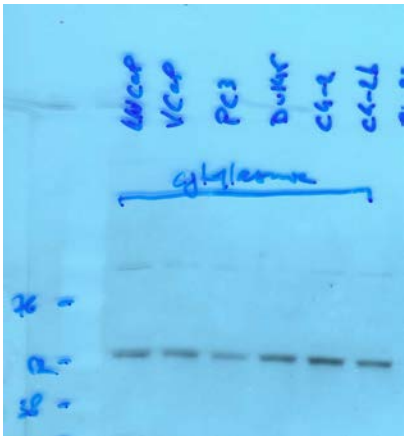

ARRB1

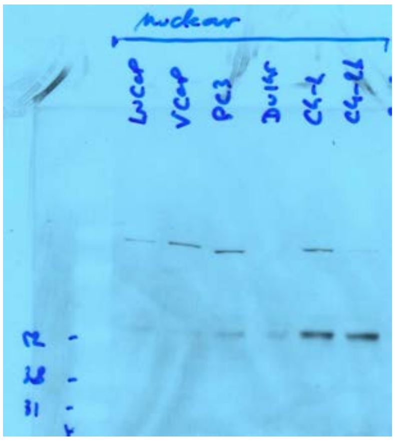

ARRB1

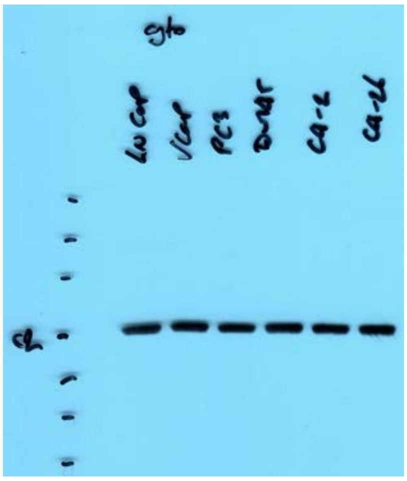

TUBB

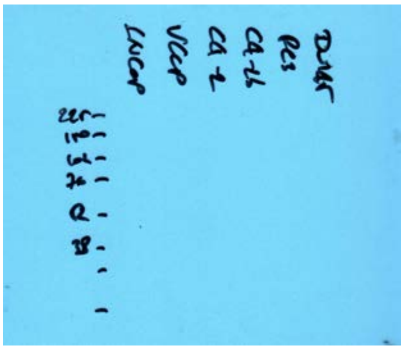

TUBB

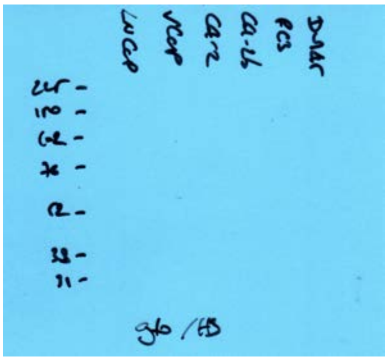

Histone H3

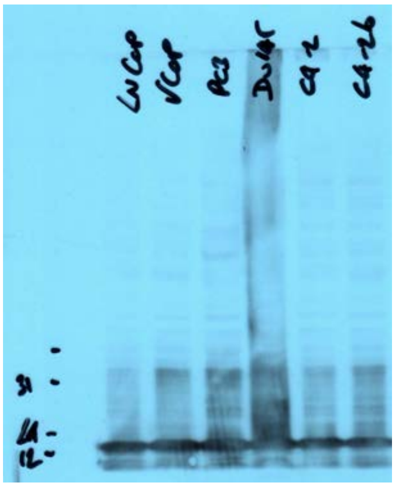

Histone H3
